# Potential effects of climate change on the distribution range of the main silicate sinker of the Southern Ocean

**DOI:** 10.1002/ece3.1138

**Published:** 2014-07-22

**Authors:** Stefan Pinkernell, Bánk Beszteri

**Affiliations:** Alfred-Wegener-Institut Helmholtz-Zentrum für Polar- und MeeresforschungAm Handelshafen 12, 27570, Bremerhaven, Germany

**Keywords:** Diatom, *Fragilariopsis kerguelensis*, Maxent, phytoplankton, Southern Ocean, species distribution model

## Abstract

*Fragilariopsis kerguelensis*, a dominant diatom species throughout the Antarctic Circumpolar Current, is coined to be one of the main drivers of the biological silicate pump. Here, we study the distribution of this important species and expected consequences of climate change upon it, using correlative species distribution modeling and publicly available presence-only data. As experience with SDM is scarce for marine phytoplankton, this also serves as a pilot study for this organism group. We used the maximum entropy method to calculate distribution models for the diatom *F. kerguelensis* based on yearly and monthly environmental data (sea surface temperature, salinity, nitrate and silicate concentrations). Observation data were harvested from GBIF and the Global Diatom Database, and for further analyses also from the Hustedt Diatom Collection (BRM). The models were projected on current yearly and seasonal environmental data to study current distribution and its seasonality. Furthermore, we projected the seasonal model on future environmental data obtained from climate models for the year 2100. Projected on current yearly averaged environmental data, all models showed similar distribution patterns for *F. kerguelensis*. The monthly model showed seasonality, for example, a shift of the southern distribution boundary toward the north in the winter. Projections on future scenarios resulted in a moderately to negligibly shrinking distribution area and a change in seasonality. We found a substantial bias in the publicly available observation datasets, which could be reduced by additional observation records we obtained from the Hustedt Diatom Collection. Present-day distribution patterns inferred from the models coincided well with background knowledge and previous reports about *F. kerguelensis* distribution, showing that maximum entropy-based distribution models are suitable to map distribution patterns for oceanic planktonic organisms. Our scenario projections indicate moderate effects of climate change upon the biogeography of *F. kerguelensis*.

## Introduction

**Figure 1:**
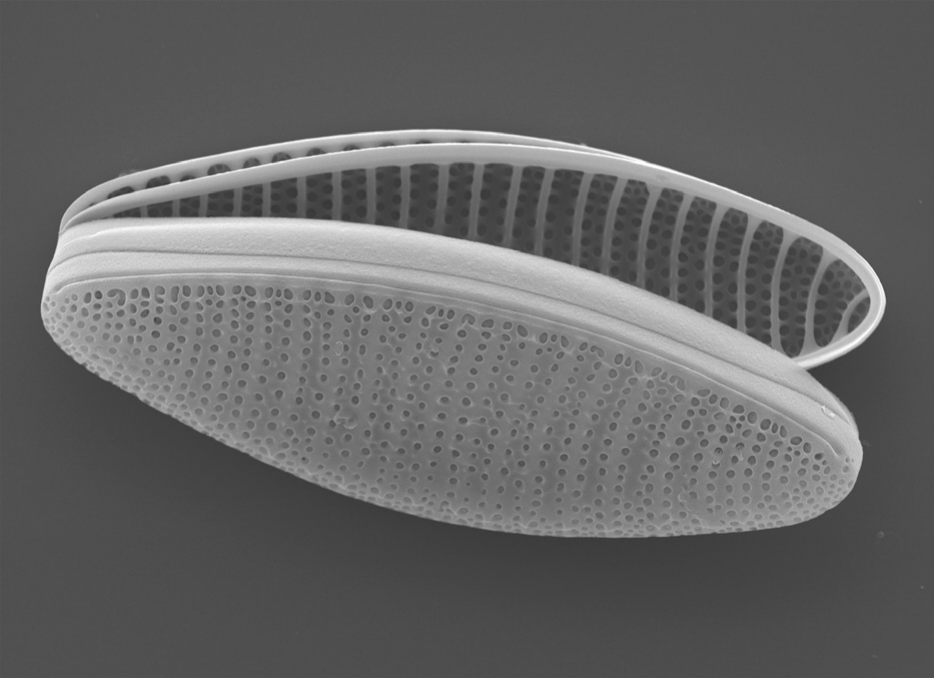
Scanning electron microscopic image of *Fragilariopsis kerguelensis* valves (image by Friedel Hinz, AWI).

Diatoms are the most important group of primary producers in the Southern Ocean, with *Fragilariopsis kerguelensis* (Fig. 1) being one of the dominant species occurring throughout the Antarctic Circumpolar Current (ACC) (Queguiner et al. 1997; Mohan et al. 2011; Lee et al. 2012). Due to its dominance in the water column and high sinking rate into the underlying siliceous ooze belt, *F. kerguelensis* contributes significantly to the removal of silicate from surface waters of the Southern Ocean (Zielinski and Gersonde 1997; Smetacek 1999; DeMaster 2002; Smetacek et al. 2012), leading to the low silicate concentrations observed at lower latitudes (Sarmiento et al. 2004). Hence, *F. kerguelensis* is often seen as a keystone species of the region, and its fate might have far-reaching consequences for global biogeochemical cycles, mainly for the silicate cycle.

Coupled global biogeochemical ocean and climate models have been applied to assess the potential consequences of climate change on diatoms (Bopp et al. 2005). Their results indicate that diatom productivity will decrease during the upcoming decades, leading to changes in ecosystem structure and the efficiency of the biological pump. However, the broad diversity of diatoms is reduced to one parameter set in such models. Diatom taxa have a wide range of variation both in their ecophysiological traits which determine their distribution and population dynamics and in their elemental composition and sinking rates, leading to widely differing impacts on biogeochemistry. Consequently, a complementary approach focusing on potential climate change responses of individual species can provide additional insights to those available from biogeochemical modeling. To date, little is known about the potential consequences of climate change upon the distribution of individual diatom species, including *F. kerguelensis*.

Occurrence of *F. kerguelensis* was reported from numerous phytoplankton surveys (Burckle and Cirilli 1987; Lee et al. 2012) as well as from sediment cores (Zielinski and Gersonde 1997; Crosta et al. 2005; Esper et al. 2010). A synthesis of data across multiple surveys and quantification and mapping of the biogeographic range of this species has not yet been attempted. In order to address this gap, and to get first insights into how climate change might affect the distribution range of *F. kerguelensis*, we applied species distribution modeling to a compilation of publicly available *F. kerguelensis* occurrence records from the plankton.

Species distribution modeling (SDM) is a commonly used approach in ecological-biogeographic research and to assess potential effects of climate change upon species distribution ranges. The technique is attractive for our purpose because, as opposed to process models, SDM does not require parameterization of population behavior and dynamics for our target organism. SDMs are capable of making use of the simplest types of distributional data, that is, presence-only records of occurrence, and of correlatively linking environmental and oceanographic factors to species distribution patterns. It is thus possible to project suitable habitats, as estimated using SDM methodology, upon future oceanographic scenarios and thereby assess potential biogeographic consequences of climate change. Differently from freshwater lake phytoplankton (Verleyen et al. 2009), distribution of oceanic phytoplankton organisms, and, in particular, of diatoms, has been shown to be more strongly affected by environmental parameters such as nutrients (nitrate, nitrite, phosphate, silicate) and oceanographic parameters (temperature, salinity), determining the water masses than by dispersal limitation (Cermeno and Falkowski 2009; Cermeno et al. 2010; Chust et al. 2013). The correlative approach of SDM seems promising to capture this effect and is thus expected to provide realistic distribution range assessments for planktonic diatoms.

Experience with species distribution modeling of marine planktonic (micro-)organisms is scarce (Sorte et al. 2010; Robinson et al. 2011), although SDM itself has been used for decades and is now a standard tool in terrestrial biogeography (Elith and Leathwick 2009). SDM studies in the marine realm are much scarcer, and their majority focused upon vertebrates (mainly fish and mammals); macro algae and invertebrates received less attention (Robinson et al. 2011), and for plankton organisms, hardly any experience is available (Weinmann et al. 2013). Concerning diatoms, SDM has been applied to forecast the potential spread of the invasive freshwater species *Didymosphenia geminata* (Kumar et al. 2009). Although this is the SDM study performed with the most closely related organism to our target species, there are huge differences in the particular habitat types (freshwater benthic vs. pelagic open ocean) between both species. Accordingly, our study is also a pilot study about the applicability of SDM methodology for studying distribution of open-ocean planktonic microorganisms and modeling habitat changes for future climate scenarios (Beaumont et al. 2008). We address several issues including choice of modeling methodology, availability, quantity, and spatial and environmental bias of observation data in public repositories, choice of oceanographic explanatory variables, and the effects of seasonality. We would like to emphasize, following the intensive debate in recent literature (McInerny and Etienne 2012a,b,c), that our primary interest here is in modeling potential distribution ranges and not niche parameters which are expected to be difficult to obtain for our target species due to strong correlations among several relevant environmental parameters throughout the Southern Ocean. For clarification, under distribution model, we will refer to a statistical model describing a relationship between environmental variables and taxon occurrences (presence-only data); whereas under distribution range, we will refer to the (observed or modeled) spatial extent of the area occupied by a particular taxon. Under distribution patterns, in this study, we also understand the temporal (for instance, seasonal) changes of this distribution range. Under projection, we will refer to displaying a distribution model output in a map to show potential distribution ranges.

## Data and Methods

### Environmental data

All models are based on four environmental parameters, selected based on availability (both for present conditions and future scenario model outputs) as well as ecological relevance: sea surface temperature, salinity, and nitrate and silicate concentrations. We also considered phosphate and iron concentrations, mixed layer depth, photosynthetically active radiation, pH, and sea-ice concentration. These were found to show hardly any influence in distribution models of *F. kerguelensis* or were not available in both data products describing present-day oceanographic conditions and in climate model outputs. Source of the datasets for current values is the World Ocean Atlas 2009 (Garcia et al. 2009; Antonov et al. 2010; Locarnini et al. 2010). For model construction, we used both yearly and monthly average values from this dataset. Future projections are based on the outputs of five climate models, which provide the full set of environmental variables used: CESM1-BGC (Long et al. 2013), IPSL-CM5A_LR (Dufresne et al. 2013), MPI_ESM_LR (Giorgetta et al. 2013), NorESM1-ME (Tjiputra et al. 2013), and HadGEM2-ES (Jones et al. 2011; Martin et al. 2011). For this study, we chose the RCP4.5 (see supplements) and RCP8.5 scenarios, where radiative forcings of 4.5 W m^−2^ (∼650 ppm CO2 equivalent) and 8.5 W m^−2^ (∼1370 ppm CO2 equivalent) are expected, respectively, for the year 2100 (Moss et al. 2010; van Vuuren et al. 2011).

All WOA layers have the same resolution (1 × 1°), extent, units (temperature in °C, nutrients in μmol L^−1^, salinity in PSU), and coordinate system (Gauss Krüger Coordinate System, GCS_WGS_1984). Climate model outputs were regridded, and measurement units were harmonized with those available in WOA.

### Observation data

**Figure 2:**
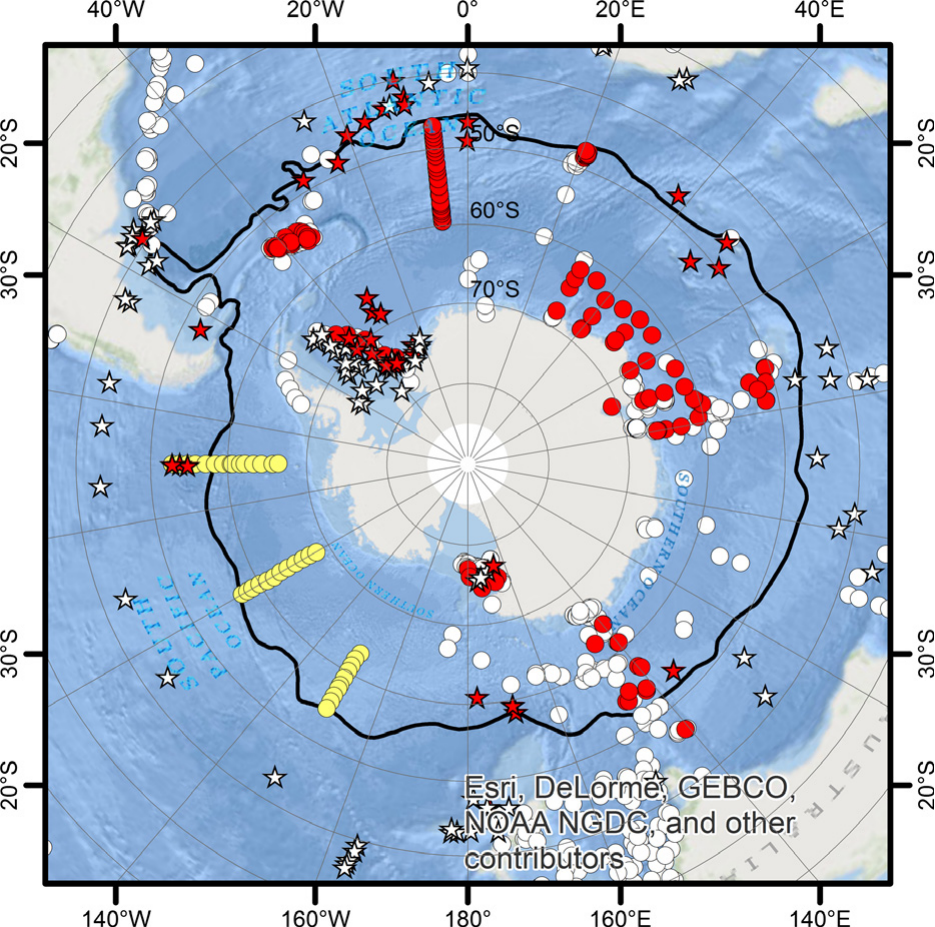
Observation data for *Fragilariopsis kerguelensis*. Red dots: presence locations based on GBIF or Global Diatom Database; yellow dots: transects of the Brategg expedition (Hasle 1969); white dots: locations of not *F. kerguelensis* diatom records from GBIF; stars: locations at which *F. kerguelensis* was found (red) and not found (white) on slides from the Hustedt Diatom Collection (BRM). Note that in this study, only the *F. kerguelensis* presence records were analyzed.

Observation data (presence-only) for *F. kerguelensis* were retrieved from two public databases: GBIF (http://www.gbif.org) and the Global Diatom Database (Leblanc et al. 2012). GBIF entries were obtained on 31 July 2013 from data resources with the following IDs: 318, 8383, 1769, 8388, 8387, 1916, 13094, and 13095 (Conkright et al. 2002; Kopczyńska et al. 2007; Trull and Bray 2012). This compilation is referred to as dataset A throughout this study. For dataset B, observations from three transects at 90°W, 120°W, and 150°W were used additionally, combined from a station list published in (Hasle 1969) and a map showing occurrence of *F. kerguelensis* at these stations (Balech 1968). After calculating distribution models for these datasets, the collection of the Hustedt Diatom Study Centre was searched for samples from areas of disagreement between different algorithms: especially, the area directly north of the inferred northern distribution boundary, as well as the Weddell Sea and the Ross Sea. Microscopic slides from samples from these areas were screened using 400× magnification for *F. kerguelensis* valves. We will refer to the final observation dataset containing also these additional observations as dataset C. Dataset A contained 129 presence entries, dataset B 166, and dataset C 210. Locations and sources of the observation data are shown in Fig. 2. The full observation dataset C (reduced to 157 points, due to identical sampling locations visited in multiple months) was used for constructing the model based on yearly averages of environmental parameters.

### Distribution models

To compare different methods for distribution modeling, we used OpenModeller (v1.3.0), an open framework implementing a broad variety of algorithms (Morin and Thuiller 2009; Souza Muñoz et al. 2009), and compared the following algorithms: Artificial Neural Network, Bioclim, Climate Space Model, Ecological-Niche Factor Analysis, Environmental Distance (4 distance metrics: Euclidian, Mahalanobis, Manhattan/Gower, Chebyshev), Envelope Score, GARP (DesktopGARP- and OpenModeller-Implementation, each with and without best subsets), Niche Mosaic, Random Forests and Support Vector Machines (SVM). Altogether, we constructed 16 distribution models with OpenModeller. For comparing models, the projection for each was thresholded at a value of 0.2, and for each grid cell, the number of models indicating presence of the species after this thresholding was displayed in a map. For final model construction and projections, we used the maximum entropy method as implemented in Maxent version 3.3.3k (Phillips et al. 2004, 2006; Phillips and Dudik 2008; Elith et al. 2011), using a beta multiplier of 1 and hinge features only. We calculated two models using the maximum entropy method: the first built on yearly averages of environmental data from WOA. For the second (monthly) model, environmental data for each observation record were extracted from the monthly dataset of its particular sampling month. For comparison, both models were projected upon the yearly averaged set of environmental data. We used the area under the curve of the receiver operating characteristic (AUC-ROC), shapes of the response curves, Jackknife tests (all with replicates using cross-validation), and consistency with background knowledge for model evaluation and comparison.

The models built using monthly environmental data were also projected on monthly environmental conditions to allow an appreciation of the seasonality of the distribution range of *F. kerguelensis*. Furthermore, these (monthly resolved) models were projected on future environmental conditions expected for the year 2100 (i.e., the five climate models listed under Environmental data), again for environmental conditions for each month of the year. In order to capture variation between differing modeling methods and starting conditions used to generate the environmental datasets, the model was projected on each of the five different climate model outputs. Means and standard deviations of these projections were displayed in maps, for each monthly average from WOA and for the RCP4.5 (see supplements) and the RCP8.5 scenario. To study effects of different sets of taxon observation records upon SDM inferences, this was carried out with all three observation datasets.

All maps were projected to South Pole Lambert Azimuthal Equal Area projection using the original color scheme of Maxent (except the algorithm consensus map in Fig. 2C). Average position of the Subantarctic Front, plotted in some maps, was obtained from http://gcmd.nasa.gov/records/AADC_southern_ocean_fronts.html (Orsi et al. 1995).

## Results

**Figure 3:**
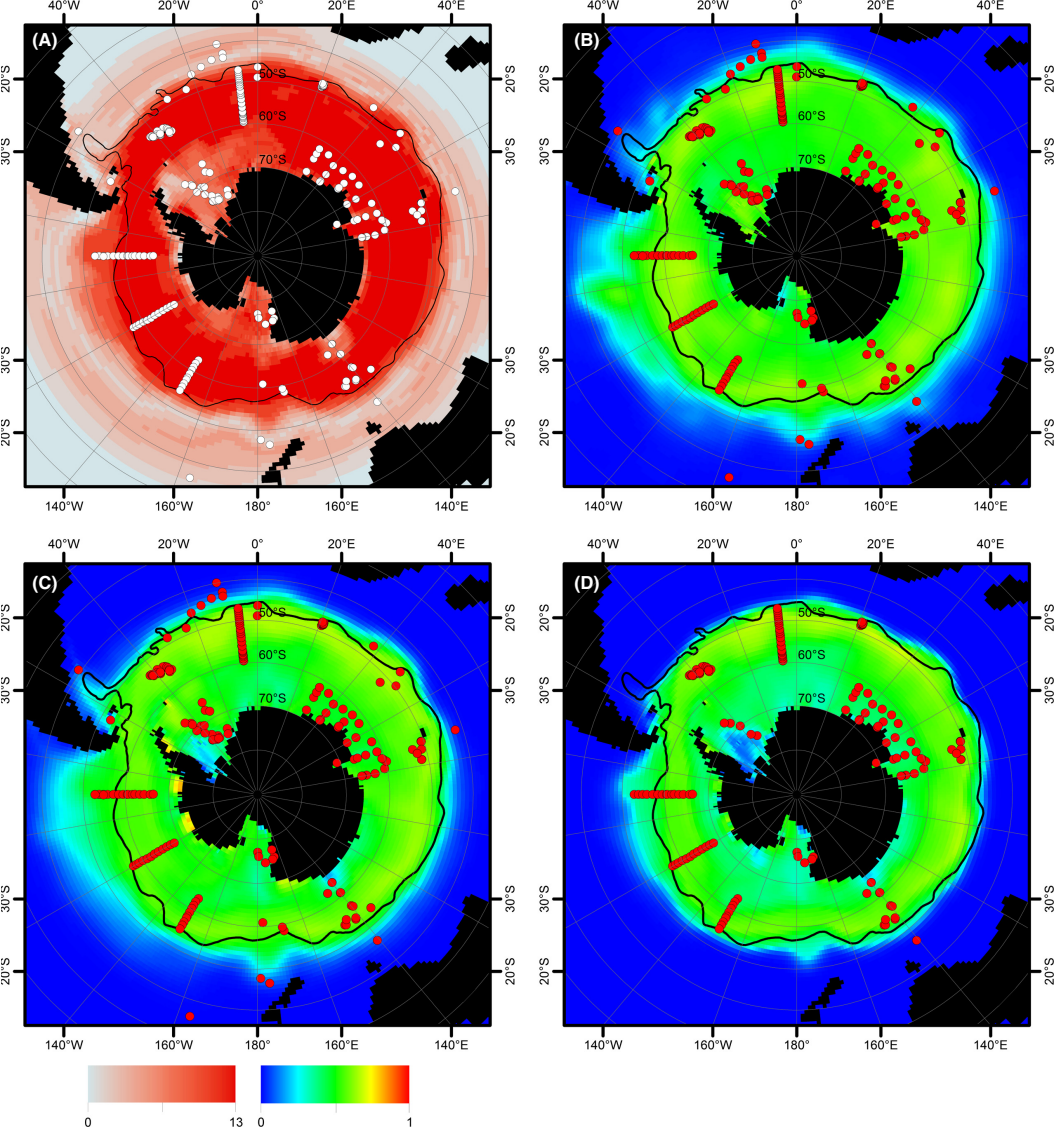
Modelled distribution of *Fragilariopsis kerguelensis* as projected on current yearly averaged environmental conditions. (A) consensus plot of 16 yearly models (calculated by different algorithms using yearly averages, see Methods for details) showing the number of models where a threshold of 0.2 was reached; (B) model based on the full dataset C calculated using yearly averages of environmental variables; (C) model based on the full dataset C calculated using monthly averages of environmental variables; (D) model based on observation dataset B calculated using yearly averages of environmental variables. Average position of the Subantarctic Front is shown by a black line. Dots represent the locations of the presence-only observation records.

For algorithm comparison, 16 distribution models were constructed with different methods using yearly averages of environmental data. These 16 models were compared with models constructed with the maximum entropy method (Maxent) using yearly and monthly environmental datasets for each of the three observation datasets (Fig. 3; we will refer to these models as yearly and monthly Maxent models, also naming the dataset the model is based on, respectively). The consensus map (Fig. 3A) shows the consensus distribution range of *F. kerguelensis* from the 16 different models calculated for method comparison. Projections upon current yearly averaged environmental conditions of the algorithm consensus (Fig. 3A), of the yearly Maxent model (Fig. 3B), and of the monthly Maxent model (Fig. 3C) all showed the northern boundary of the potential distribution range near, but not exactly following, the Subantarctic Front (SAF). An exception was the eastern section of the Pacific Ocean, where this boundary was located further northward from the SAF. Models based on the full observation dataset (dataset C) show a slightly northward shifted northern boundary (Fig. 3A–C) compared with models based only on publicly available observations (dataset B, Fig. 3D). The projections of the monthly Maxent models (Fig. 3C and D) both showed a gap in the potential distribution in the southern Weddell Sea, which was not present in the yearly Maxent model projection (Fig. 3B). Some observation points in the region between Australia and Antarctica and in the Atlantic sector of the Southern Ocean were located outside the projected potential distribution ranges. In all other regions, observation points fell within the potential distribution ranges estimated using all methods. The algorithm consensus plot (Fig. 3A) shows that differences among algorithms in the estimated potential distribution area were mainly located south of 60°S, especially in the region affected by the Weddell Gyre, as well as in the belt north of the main predicted distribution area up to 30°S. The emerging pattern, that is, the main distribution range of *F. kerguelensis* being the Antarctic Circumpolar Current, is consistent with literature reports.

The maximum entropy method had similar results to the consensus of numerous different modeling algorithms. It provides a coherent toolset for niche modeling and projection, also allowing the use of multiple environmental data layers per variable for model construction. Because of this consistency and good experiences in the literature (Townsend Peterson et al. 2007), all further analyses about seasonality and for future projections were performed using the maximum entropy method.

**Figure 4:**
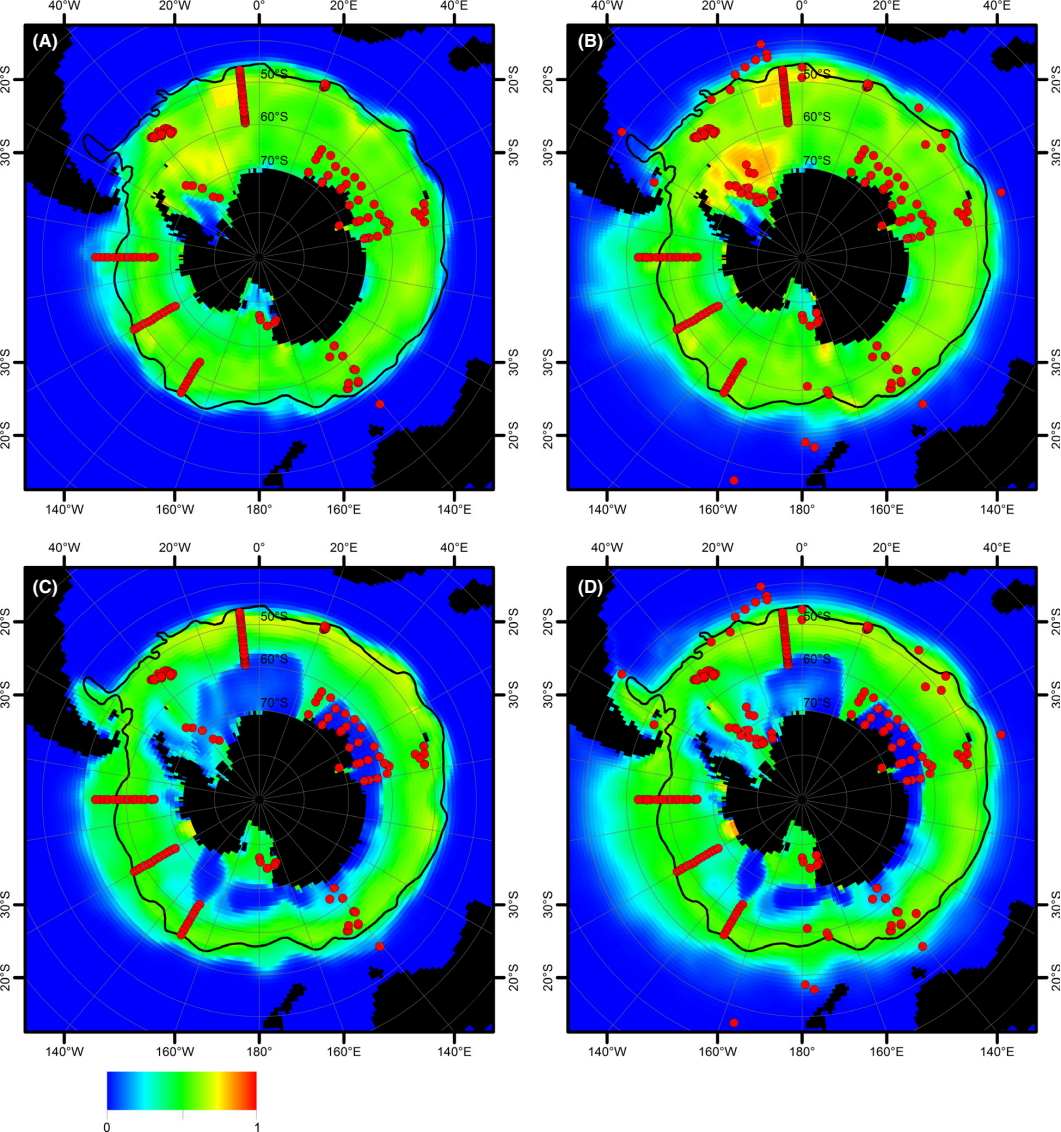
Monthly distribution models of *Fragilariopsis kerguelensis* (based on dataset B – to the left and dataset C – to the right) as projected on World Ocean Atlas data for current summer (February, upper half) and winter (August, lower half) conditions. (A) February, dataset B; (B) February, dataset C; (C) August, dataset B; (D) August, dataset C. Red dots represent the presence-only observation records upon which the model is based. Average position of the Subantarctic Front is shown by a black line.

Projections of the monthly Maxent model (dataset C) on current summer and winter environmental conditions (Fig. 4) agreed well with the projection on yearly averaged data (Fig. 3), although the former indicated some seasonal differences (for projections for all 12 months, see Figs. S1, S4, and S7). The models based on dataset B (Fig. 4A and C) only slightly differed from those based on dataset C (Fig. 4B and D), mainly in the east pacific sector of the Southern Ocean. For datasets B and C, the area between South America and the Falkland Plateau appeared as part of the potential distribution range only in the winter projection of the monthly Maxent models (although some other methods indicated this area to be part of the potential distribution range of *F. kerguelensis*, see Fig. 3A). The northern boundary of the distribution range roughly followed the SAF, except for the eastern part of the Pacific sector of the Southern Ocean: Similarly to the yearly models, the northern range boundary was located further north from the SAF. Toward the Antarctic continent, only small gaps in the potential distribution range of *F. kerguelensis* were found in the Weddell Sea and the Ross Sea under summer conditions. Projection of the monthly Maxent model on winter environmental conditions resulted in a different potential distribution map (Fig. 4B). The northern boundary of the distribution was similar to the other projections except for a bulge at 60°W, around the Falkland Islands, with its northern tip at 40°S. The southern boundary of the distribution range was shifted northwards, to near the southern boundary of the ACC, except for the Ross Sea which was still part of the potential distribution area.

Although both Maxent models (yearly and monthly) based on the full observation dataset C were built on the same set of “raw” observation data, 210 presence records were used for the training of the monthly model, but only 157 records for the yearly model. This is explained by multiple sampling events at identical positions in different months. Such observations were counted as duplicates for the yearly averages model, whereas they represented distinct observation records (with distinct values for their associated environmental variables) for the monthly model. The yearly model reached an AUC-ROC value of 0.902. The monthly model performed slightly better, reaching 0.923. In both models, nitrate was the most influential variable (96.3% and 88.5%, respectively, in the yearly and monthly models). Relative contributions of the other variables to the models differed. Sea surface temperature became more important in the monthly model with a contribution of 7% in contrast to only 2% in the yearly model. In the monthly model, the oceanographic variables salinity and silicate together made up 4.6%, in contrast to only 1.7% for sea surface temperature and no effect of salinity in the yearly model.

**Figure 5:**
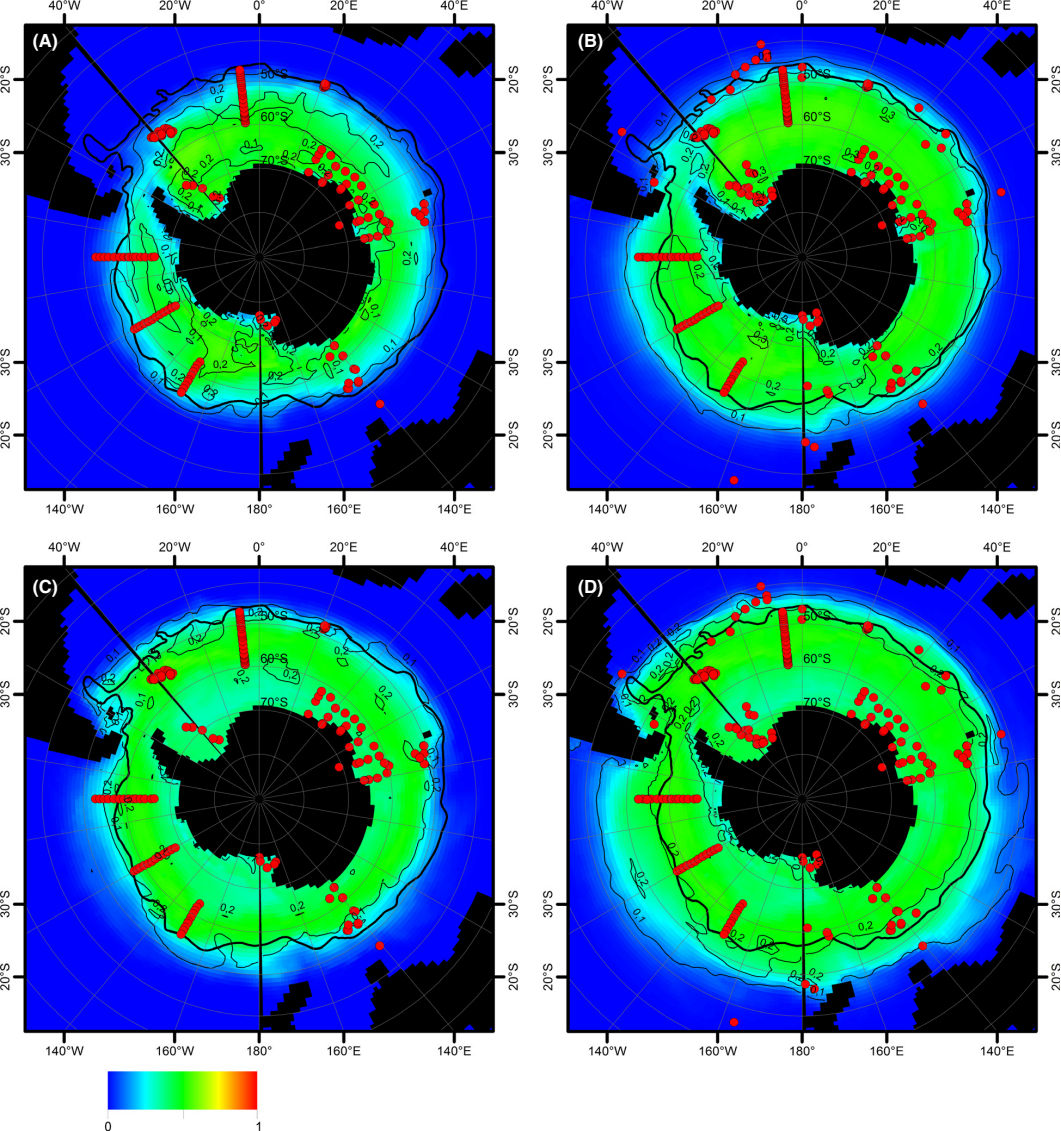
Potential distribution of *Fragilariopsis kerguelensis* (monthly model): mean (by color) and standard deviation (as black contour lines) of projections to five different climate model outputs. (A) RCP 8.5 scenario, February 2100, based on dataset B; (B) RCP 8.5 scenario, February 2100, based on dataset C; (C) RCP 8.5 scenario, August 2100, based on dataset B; (D) RCP 8.5 scenario, August 2100, based on dataset C. Red dots represent the presence-only observation records upon which the model is based. Average position of the Subantarctic Front is shown by a black line.

Projections of the monthly Maxent model based on datasets A and B on summer and winter environmental conditions modeled for the year 2100 (Fig. 5) showed a decreased distribution area, along with an overall decrease in Maxent scores throughout (projections for all 12 months are provided in Figs. S2, S5, and S8 for RCP4.5 and S3, S6, and S9 for RCP8.5).

Whereas a cutoff selection between 0.1 and 0.3 of Maxent's logistic output score would hardly affect the present-day northern distribution boundary of *F. kerguelensis* (Fig. 4), this choice makes a bigger difference in the case of future scenarios. At a cutoff of 0.3, the northern range boundary shifts poleward to south of the polar front in the RCP8.5 (Fig. 5A–C) scenario. For dataset B, at a cutoff of 0.1, the northern distribution boundary would be inferred close to the northern range boundary for present-day conditions for the winter (Fig. 5C), but would still substantially shift to the south in the summer (Fig. 5A). In contrast, the northern boundary inferred for summer conditions lies close to the Subantarctic Front (and thus the current position of the northern range boundary) for the Atlantic and Indian Ocean sector of the Southern Ocean for dataset C. In the Pacific sector, this boundary again depends on the threshold value ranging from 52°S at threshold of 0.3 up to roughly 46°S at 0.1 (Fig. 5B). For winter conditions, threshold selection has an even higher impact on the location of the distribution boarder ranging from 41°S to 47°S (threshold 0.1 and 0.3) in the Pacific sector. Because we have no meaningful criterion for selecting a cutoff value, the uncertainty in the northern boundary of the future distribution range of *F. kerguelensis* remains substantially higher than for the current distribution. The lower Maxent logistic scores/habitat gaps observed in the austral winter for current conditions were not present in any of the scenario projections in any season.

Projections of the monthly Maxent model based on dataset C show different future distributions: Compared with current summer distribution, this model shows an only slightly decreased distribution area. For the winter conditions, the northern boundary does not shift to the south, as in the models based on datasets A and B, but remains close to its current position or even shifts slightly to the north in the Atlantic sector.

Notably, several projections on future environmental conditions showed a gap in the potential distribution area around and in the continuation of the Antarctic Peninsula, most pronouncedly in the case of summer RCP8.5 (Fig. 5A and B). The underlying pattern in the environmental datasets is lower silicate and nitrate concentrations and lower salinity than in the neighboring areas. The CESM1-BGC model predicted substantially lower nitrate concentrations for the end of this century in the Southern Ocean compared to the other four GCMs, and this also resulted in substantially reduced projected distribution areas for *F. kerguelensis*. However, this had only negligible impacts on the mean distribution area over the five GCMs considered (see Fig. S15 for a comparison).

## Discussion

In this study, we present the first quantitative models characterizing the current distribution range and forecasting potential range shifts for the main silica sinker of the world ocean, the diatom *F. kerguelensis*. Due to the scarcity of experience with distribution modeling of plankton organisms, we first compared numerous modeling algorithms and found that both the consensus of several different modeling algorithms and the maximum entropy method estimated similar potential distribution ranges which were also in accord with previous literature reports and background knowledge. Taking into account sampling date and using monthly (as opposed to annual) average values of environmental parameters, we could calculate seasonally resolved distribution models and range projections. These monthly models incorporate the strong seasonality effects at these high latitudes and can be expected to be more accurate representations of distribution patterns than those calculated based on yearly averages. Nonetheless, the seasonal–spatial coverage of observations that would be required to validate this assumption is currently not available. The fact that most sampling campaigns at these high latitudes take place in the austral summer leads to a strong seasonal bias in the dataset. Another issue of importance for seasonal models is that sampling date is not available for all database entries. For *F. kerguelensis*, the number of observations containing date information was sufficient for producing meaningful model outputs. Besides the loss of precision in environmental data brought about by the use of yearly averages, yearly models also discard potentially useful information in the case of seasonally distinct samples from the same or nearby locations. The 210 entries used for the seasonal model resulted in only 157 distinct entries for the yearly model due to multiple samples taken at different times of the year at nearly identical locations. For many other phytoplankton taxa, public availability of georeferenced taxon observations is much worse than for our target species and currently limiting for the application of species distribution modeling methodology.

The AUC-ROC values were high in all models (yearly models: dataset B: 0.933, dataset C: 0.902; monthly models: dataset B: 0.94, dataset C: 0.923). These uncommonly high values can be explained by the relatively small distribution area of *F. kerguelensis* and the distinct oceanographic characteristics of the Southern Ocean, compared with the sampling area of the background points covering the entire world oceans. Although our high AUC-ROC values are difficult to compare with other studies, they are appropriate for comparing similar models among each other and indicate a somewhat superior performance of the monthly models over the yearly ones.

Besides public availability and spatial and seasonal spreads, taxonomic accuracy of taxon observation records is also crucial for model quality. When using GBIF and GDD data, we had to rely on identifications of the data sources as they do not provide voucher images that would enable taxonomic cross-checking. We expect this not to be problematic for our target taxon as it can easily be identified even with little experience, but this does not hold to marine diatom and phytoplankton taxa in general. A few locations from the public databases could be validated by cross-checking of associated microscopic slides of the Hustedt Collection – notably a full set of slides from the Pacific transects of the Brategg expedition which are reported about in (Hasle 1969) and (Balech 1968) are available here. Surprisingly, evaluation of harvesting of different data sources (data not shown) showed that part of the observation data is apparently not shared among three interconnected data providers GBIF, OBIS (http://www.iobis.org), and PANGAEA (http://www.pangaea.de). This issue needs to be circumvented for taxa where few observational records are available through querying of different data sources and cleaning the data of duplicates.

Sampling bias turned out to be a rather complex and subtle issue during this study. It is expected that even moderately sized observation datasets can be sufficient to create meaningful distribution models if the underlying data points are properly distributed. For *F. kerguelensis*, the number of entries in public databases is relatively high (at least compared to other phytoplankton taxa), but a regional bias is immediately apparent in the distribution of data points. Entries are concentrated in the Atlantic and Indian Ocean sectors of the Southern Ocean, and in the region between Australia and Antarctica, whereas the Pacific sector is underrepresented (dataset A). We could somewhat counter this bias by including observation points from three Pacific transects in dataset B from Hasle (Balech 1968; Hasle 1969). The addition of these data points resulted in no substantial changes in the inferred biogeographic range and its seasonality, which we attribute to the fact that environmental gradients are steeper in the north–south direction than along lines accompanying the circumpolar movement of the ACC water masses. Accordingly, circumpolar extrapolation of distribution ranges should be possible as long as the latitudinal environmental gradients are well sampled (to the latter aspect we return below).

Global sampling biases for marine diatom taxa obviously also exist: As a query for Bacillariophyta on the GBIF portal quickly reveals, almost all other regions of the world ocean are undersampled when compared to the North Atlantic. Nevertheless, it could be argued that sampling effort resulting in the GBIF and Global Diatom Database records is well distributed across the world ocean at least at a broad geographic scale of relevance for the spatial resolution applicable for passively floating pelagic organisms like planktonic diatoms. For the genus “Fragilariopsis”, GBIF lists over 4000 entries, and over 600.000 entries for the phylum “diatoms” (Bacillariophyta), distributed worldwide over the oceans. To probe the effect of this global bias upon our results, we constructed a bias grid reflecting the spatial distribution of these diatom entries from GBIF and calculated a Maxent model incorporating this information. The inferred distribution range hardly differed from those resulting from models without the bias grid, further supporting that the heterogeneity in sampling effort did not substantially affect our results (see Fig. S10). In spite of this, closer inspection revealed a more subtle sampling bias, which had a pronounced effect upon distribution range projections for future oceanographic scenarios.

When looking at the distribution of all diatom observation records from Southern Hemisphere marine locations (Fig. 2), it became apparent that around the northern edge of the ACC and just north of it very few diatom observations are available. It appears that most phytoplankton sampling effort in the Southern Hemisphere is targeted at the Antarctic/Subantarctic regions, and most of these expeditions do not sample north of around 40°S. Thus, the question arose if the *F. kerguelensis* observations from GBIF and GDD covered the real northern distribution boundary of the species, and if this sampling bias influenced our inferences about its distribution range. Therefore, we searched the Hustedt Diatom Collection (BRM) for microscopic slides originating from locations around and just north of the northern boundary of the inferred distribution area based on datasets A and B and scored these for the presence of *F. kerguelensis* valves (dataset C). This analysis revealed that the species indeed can be found somewhat further north than the publicly available observation points suggest, even if mostly at low abundances. The addition of these data points to the SDM input (dataset C) led to only a minor northward shift of the inferred northern distribution boundary, now located somewhat north of the Subantarctic Front. Its effects upon scenario projections were, however, more substantial. These still indicated a change in seasonality (increasing habitat suitability in the southern coastal areas in the winter), and we could generally observe decreased values of the Maxent logistic output throughout the distribution area when compared to the current distribution. The southward shift projected for the northern distribution boundary of the species, however, became smaller and limited to the summer.

The main environmental drivers of the distribution of *F. kerguelensis*, according to our models, are nitrate concentrations and sea surface temperatures (SST). Whereas both variables contribute to defining the inferred northern distribution boundary, the southern “boundary” (gaps in the inferred distribution area in some areas next to Antarctic continent in the winter) is mainly defined by low SST which here probably functions as a proxy to the sea ice, directly not included in our models. The overwhelming importance of nitrate, accompanied by the almost negligible influence of silicate, in defining the northern distribution boundary of *F. kerguelensis* might appear surprising at first sight because *F. kerguelensis* is known to have an atypically high Si:N ratio when compared to most other diatoms (Brzezinski 1985; Hoffmann et al. 2007), and, accordingly, has a high silicate demand. Silicate and nitrate distributions are highly correlated in the ocean, and it could be that this correlation leads to an exchangeability of the two variables in our distribution models. However, one of the notable regional exceptions from this global correlation is represented by the northern rim of the ACC, a region encompassing the northern distribution boundary of *F. kerguelensis* (see Fig. S11). Here, silicate is depleted (due to the growth of highly silicified diatoms, not the least *F. kerguelensis* itself) toward the north, while nitrate is not consumed fully and reaches concentrations which are atypically high for water masses so strongly depleted of silicate. Distribution of nitrate and silicate concentrations at the observation locations indeed reveals that whereas *F. kerguelensis* is rarely observed at low nitrate concentrations, many observation points fall into silicate-depleted locations (Fig. S14). Thus, although paradoxical at first sight, it appears that sea surface distribution of nitrate is more informative about the biogeography of this highly silicified diatom than silicate is.

Whereas the position of the inferred northern distribution boundary proved insensitive to the addition of observation points from the South Pacific, it did respond to the addition of records from around the northern edge of the ACC. This is less surprising than it might seem upon first sight, as the South Pacific observation points had rather similar environmental characteristics to those already in the dataset from the South Atlantic (Fig. S14), but the northernmost observation substantially increased the range of influential environmental variables in the dataset. In dataset B, with three exceptions, all observations came from locations with a nitrate concentration >15 μmol/L and SST <7.5°C (see Fig. S14). Interactive visualization of the models based on dataset B with the Maxent explain tool also indicated that the northern distribution boundary was defined by nitrate concentrations around 15 μmol/L in these models. When adding our newly captured observation records from around the northern edge of the ACC, both SST and nitrate concentration for several data points fell beyond these limits (to below 5 μmol/L nitrate and to almost 15°C SST, apart from one extreme outlier observation for both variables, Fig. S14). According to the Maxent explain tool, the northern distribution boundary was now located at a nitrate concentration below 10 μmol/L and an SST of 10°C. The fact that future distribution range projections are affected more strongly by this change than the current distribution patterns can be explained by the differing strength of the south to north nitrate gradient today and in the oceanographic model outputs for 2100 (shown in Fig. S12). While areas of peak nitrate concentrations are projected to sink substantially in RCP8.5 compared with the current situation, areas with moderately high nitrate concentrations (between 10 and 20 mmol/L) are extending, especially in the summer. But the latitudinal nitrate gradient is projected to weaken also in the winter, as indicated by the wider spacing of μmol/L isolines in Fig. S12. If the full dataset C (including the additional records obtained from the northern ACC) is taken to be a more accurate representation of the distribution of *F. kerguelensis*, we can conclude that oceanographic changes projected for 2100 for RCP8.5 will likely not affect its distribution range substantially. On the other hand, the decreasing nutrient supply over the ACC (as shown by Fig. S12) might well lead to changes in its abundance, but addressing these would require a different approach.

We also cannot exclude from our results that SST shifts (Fig. S13) projected for the next century might affect the distribution range of *F. kerguelensis*. Although in the correlative framework used here, nitrate seems the single most important variable explaining the distribution of *F. kerguelensis*, SST and temperature tolerance might nevertheless be important mechanistic drivers of its future distribution. However, the conclusions we can draw from our analyses about temperature tolerance of this species are rather limited. Although several observation points have associated SST values > 11°C (one even reaching almost 20°C), this cannot safely be interpreted to imply that *F. kerguelensis* can survive longer periods at such temperatures. Fiala and Oriol (1990) observed the upper growing temperature for *F. kerguelensis* in a culture at 7°C. In a small experiment where we incubated three *F. kerguelensis* cultures at 4, 7, and 11°C, all cultures disintegrated and died after around one week at the highest temperature (data not shown). This does not exclude the possibility that *F. kerguelensis* can acclimate to higher temperatures when given more time (in our experiment, they were transferred to 11°C from their usual growing temperature of 4°C after 1 week acclimation at 7°C), and a more systematic investigation of the temperature tolerance of this species is needed before far-reaching consequences can be drawn.

Hasle located the northern distribution boundary of *F. kerguelensis* at 40 to 56°S (Hasle 1976). In this region, roughly also corresponding to the Subantarctic Front, a sharp increase in chlorophyll-a concentrations can be observed, marking “a rapid transition from unproductive subtropical waters to highly productive temperate and subpolar systems” (Cermeno et al. 2010). The northern boundaries of the projected ranges of the models presented in this study are usually near the SAF. The largest exception is the southwest Pacific where the northern distribution boundary in our results usually lies substantially further north than the SAF. In the southwest Pacific, we can observe less marked latitudinal gradients in environmental parameters than in the other ocean basins crossed by the ACC, probably partially caused by cold, nutrient-rich Antarctic surface waters transported by the Humboldt Current. The distance between the Subantarctic and Subtropical Fronts is also greater in this region than in the South Atlantic and South Indian Ocean (Orsi et al. 1995). It seems that these regional oceanographic features lead to a more blurred distribution boundary for *F. kerguelensis* in the South Pacific than in other regions.

In the sediment, *F. kerguelensis* was found further north, up to the Subtropical Front (STF) (Zielinski and Gersonde 1997; Crosta et al. 2005). In sediment studies, *F. kerguelensis* is reported as an endemic Southern Ocean species with a temperature range of −1 to 18°C and a “significant drop in abundance at temperatures > 13.5°C” (Zielinski and Gersonde 1997). Highest abundances can be observed in open-ocean regions, not or only weakly effected by sea ice, but in the sediment, *F. kerguelensis* was also reported to be found in regions with up to 8 month of sea-ice cover (Crosta et al. 2005). Altogether, the distribution range of *F. kerguelensis* based on sediment records appears somewhat broader than indicated by plankton records.

Environmental data in the World Ocean Atlas of the high latitudes, especially south of 70°S, are reported to be less accurate than for lower latitudes (Tyberghein et al. 2012). This cautions for care in the interpretation of the southern distribution boundaries of *F. kerguelensis*. However, the northern distribution boundary of this species is of more ecological/biogeochemical significance, and as this boundary lies north of 55°S, we expect it to be devoid of these accuracy problems. The gaps in the inferred distribution area next to the Antarctic continent should also be interpreted with caution, as water mass movements are expected to transport the species into and through these areas when it grows in neighboring regions. In terms of environmental variables, these gaps are defined by SST < −1.8°C, and these values are indicative of substantial sea-ice coverage. Although ice-covered regions are not seen as the typical habitat of *F. kerguelensis*, and the species indeed usually appears at low abundance in ice-covered areas, a large number of observation records (including several we have obtained ourselves, and for which physical vouchers are deposited in the Hustedt Diatom Collection) confirm the presence of *F. kerguelensis* under sea ice, for example, in the Weddell Sea and Ross Sea.

Our choice of explanatory variables was strongly influenced by practical considerations, that is, the availability of high-quality interpolated data products for present-day distributions and availability of the same variables in climate model outputs for future scenarios. Notably, we could not include iron availability as an explanatory variable. It is widely appreciated that iron is an important factor influencing phytoplankton growth and species distributions in the Southern Ocean (Smetacek et al. 2004; Assmy et al. 2013). However, iron concentrations are more difficult and costly to measure than macronutrients such as silicate or nitrate. Presently, no iron concentration datasets with a global coverage are available that could be used in the distribution modeling setup used in this work. Its complex biogeochemistry also makes iron distributions difficult to model, and availability of iron concentrations in biogeochemical model output is also limiting currently.

The seasonal model allows us to appreciate the seasonality in distribution patterns expected for phytoplankton taxa of high latitudes. Interestingly, the seasonality revealed by our distribution models for *F. kerguelensis* for present-day conditions differs from the seasonality prospected for the climate change scenarios. At present, the most marked seasonal change inferred by the model seems to relate to the southern distribution boundary, which moves somewhat toward north and away from Antarctica in the austral winter, whereas the northern range boundary changes little.

A notable feature of our distribution range projections upon environmental data modeled for the year 2100 is the presence of a gap with substantially lower or zero values of habitat suitability northeast of the Antarctic Peninsula (Fig. 5A and B). Inspection of the underlying environmental datasets (especially of the HadGEM2-ES outputs) reveals that in this area, water masses with lower salinity and nutrient concentrations are found than in the direct neighborhood. This effect seems to be stronger in the austral summer and might represent a melting signal, but was not previously noted or interpreted by oceanographic modeling studies. The biological/biogeographic significance of such a water band with distinct physicochemical characteristics from the rest of the ACC is difficult to assess, but in the worst case, it might lead to a population dynamic bottleneck for *F. kerguelensis* on their circumpolar drift within this current. Insofar, further investigation of this anomaly would be of ecological relevance.

The method used here, that is, correlative modeling of presence-only data, represents the simplest methodology that can be used for characterizing species distributions. As this discussion shows, the method has strong limitations both in terms of capturing mechanistic determinants of biogeography and in ecological interpretability of the results. As far as biogeography goes, the compilation of presence–absence datasets and experimental determination of tolerance limits could represent next steps to address these issues. Beyond that, modeling absolute abundances or species specific biomasses would of course be of substantially more ecological relevance than modeling distribution ranges, but it has to be noted that iron concentrations are probably more crucial determinants of *F. kerguelensis* population dynamics than they are for the distribution range of the species, and this presents the challenge of compilation of a large interpolated data product for iron concentrations similar to World Ocean Atlas. Alternatively, iron concentrations for such analyses could perhaps be obtained from biogeochemical modeling constrained by observation data.

## Supporting Information

**Figure S1.** Monthly Maxent model based on dataset A projected on maps of current environmental values from World Ocean Atlas (WOA). **Figure S2.** Monthly Maxent model based on dataset A projected on RCP 4.5 scenarios for year 2100. **Figure S3.** Monthly Maxent model based on dataset A projected on RCP 8.5 scenarios for year 2100. **Figure S4.** Monthly Maxent model based on dataset B projected on WOA. **Figure S5.** Monthly Maxent model based on dataset B projected on RCP 4.5 scenarios for year 2100. **Figure S6.** Monthly Maxent model based on dataset B projected on RCP 8.5 scenarios for year 2100. **Figure S7**. Monthly Maxent model based on dataset C projected on WOA. **Figure S8.** Monthly Maxent model based on dataset C projected on RCP 4.5 scenarios for year 2100. **Figure S9**. Monthly Maxent model based on dataset C projected on RCP 8.5 scenarios for year 2100. **Figure S10.** Yearly Maxent model based on dataset B built using a bias grid. **Figure S11.** Principal component analysis of environmental predictors from WOA. **Figure S12.** Current and predicted future distribution of nitrate (WOA and RCP 8.5 scenario for 2100). **Figure S13.** Distribution of sea surface temperature (current WOA and RCP 8.5 scenario for 2100). **Figure S14.** Distribution of environmental parameter values (SST, nitrate, and silicate) at sampling sites for the three observation datasets. **Figure S15.** Comparison of mean distribution projections upon all 5 GCMs vs. only 4 GCMs (without the outlier CESM1-BGC).
