# Supplementary material for: Potential effects of climate change on the distribution range of the main silicate sinker of the Southern Ocean
Source: Ecol Evol. 2014 Jul 22;4(16):3147–61. doi: 10.1002/ece3.1138 (PMC4222203; doi:10.1002/ece3.1138)
Supplement: Supplementary file 1 [file ece30004-3147-sd1.pdf]

Supplement

Fig. S1: Monthly Maxent model based on dataset A projected on maps of current environmental values from World Ocean Atlas (WOA)

Fig. S2: Monthly Maxent model based on dataset A projected on RCP 4.5 scenarios for year 2100

Fig. S3: Monthly Maxent model based on dataset A projected on RCP 8.5 scenarios for year 2100

Fig. S4: Monthly Maxent model based on dataset B projected on WOA

Fig. S5: Monthly Maxent model based on dataset B projected on RCP 4.5 scenarios for year 2100

Fig. S6: Monthly Maxent model based on dataset B projected on RCP 8.5 scenarios for year 2100

Fig. S7: Monthly Maxent model based on dataset C projected on WOA

Fig. S8: Monthly Maxent model based on dataset C projected on RCP 4.5 scenarios for year 2100

Fig. S9: Monthly Maxent model based on dataset C projected on RCP 8.5 scenarios for year 2100

Fig. S10: Yearly Maxent model based on dataset B built using a bias grid

Fig. S11: Principal component analysis of environmental predictors from WOA

Fig. S12: Current and predicted future distribution of nitrate (WOA and RCP 8.5 scenario for 2100)

Fig. S13: Distribution of sea surface temperature (current WOA and RCP 8.5 scenario for 2100)

Fig. S14: Distribution of environmental parameter values (SST, nitrate and silicate) at sampling sites for the three observation datasets

Fig. S15: Comparison of mean distribution projections upon all 5 GCMs vs. only 4 GCMs (without the outlier CESM1-BGC)

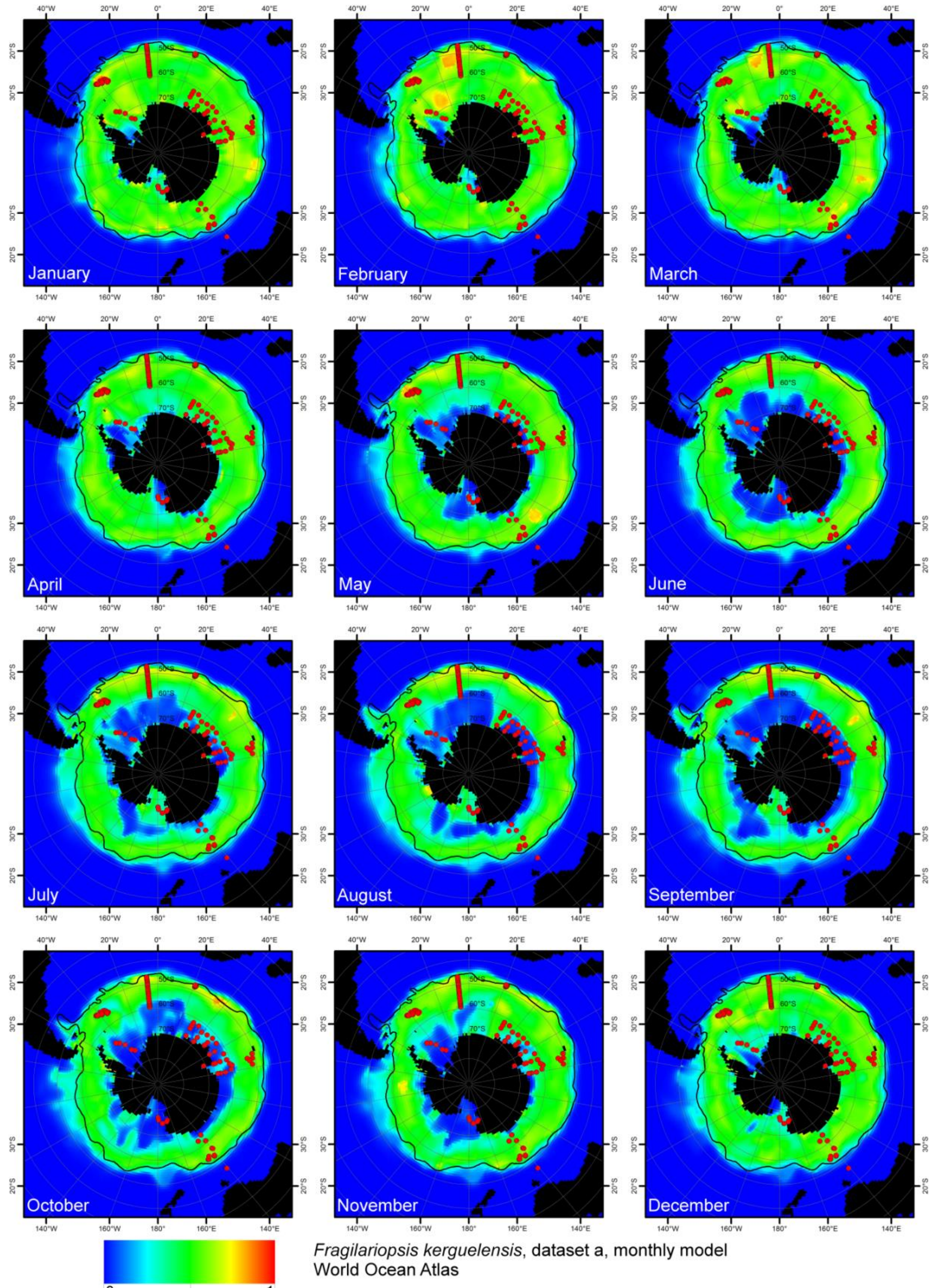

Fig. S1: Projections of the monthly Maxent model based on dataset A on monthly environmental data of the World Ocean Atlas in the course of the year. Average position of the Subantarctic Front is shown in black, observation points upon which the model is based are marked by red dots.

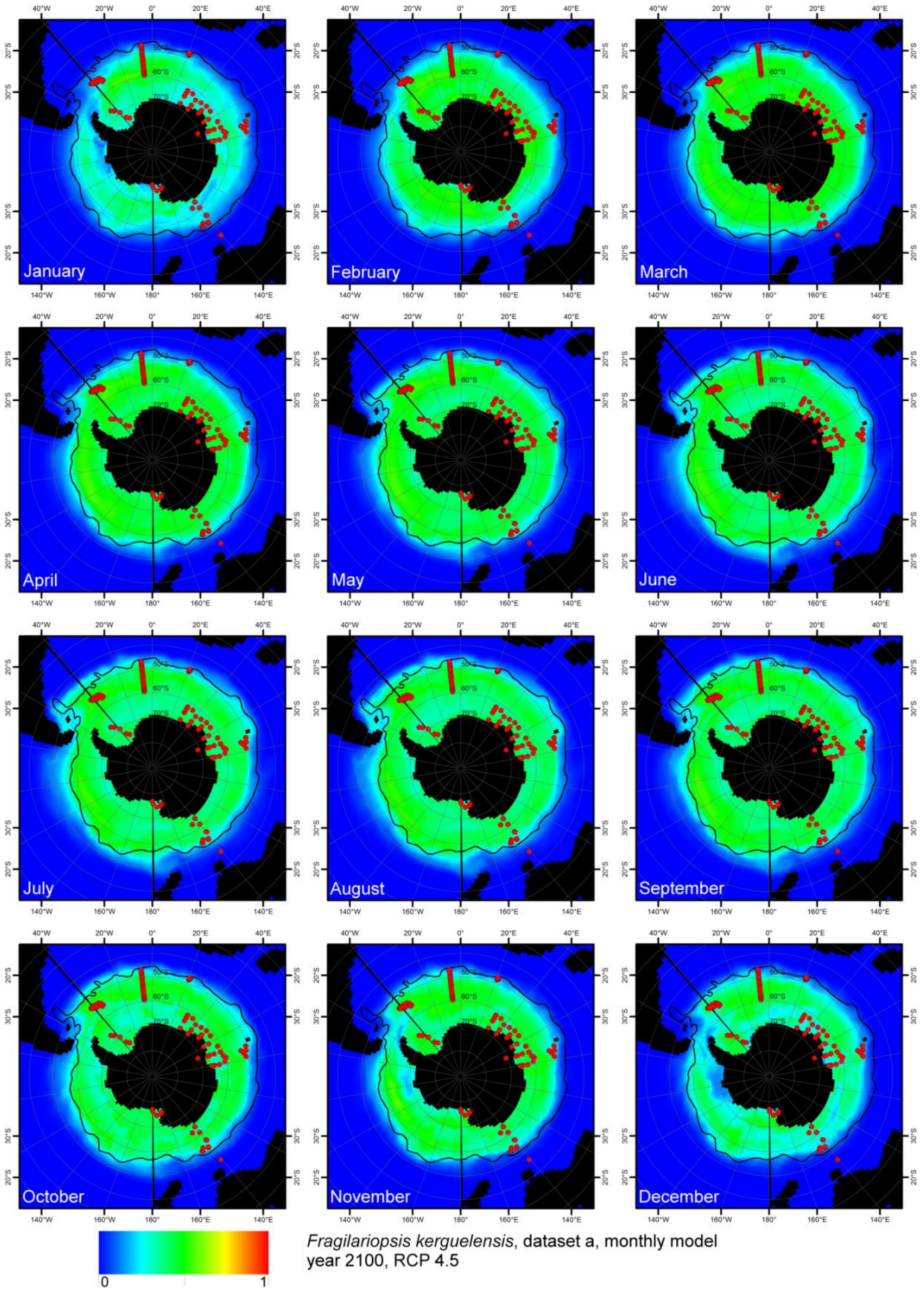

Fig. S2: Projections of the monthly Maxent model based on dataset A on environmental data predicted by climate models for the year 2100 for the RCP 4.5 scenario. The plots show the mean of the projections to five different climate model outputs. Average position of the Subantarctic Front is shown in black, observation points upon which the model is based are marked by red dots.

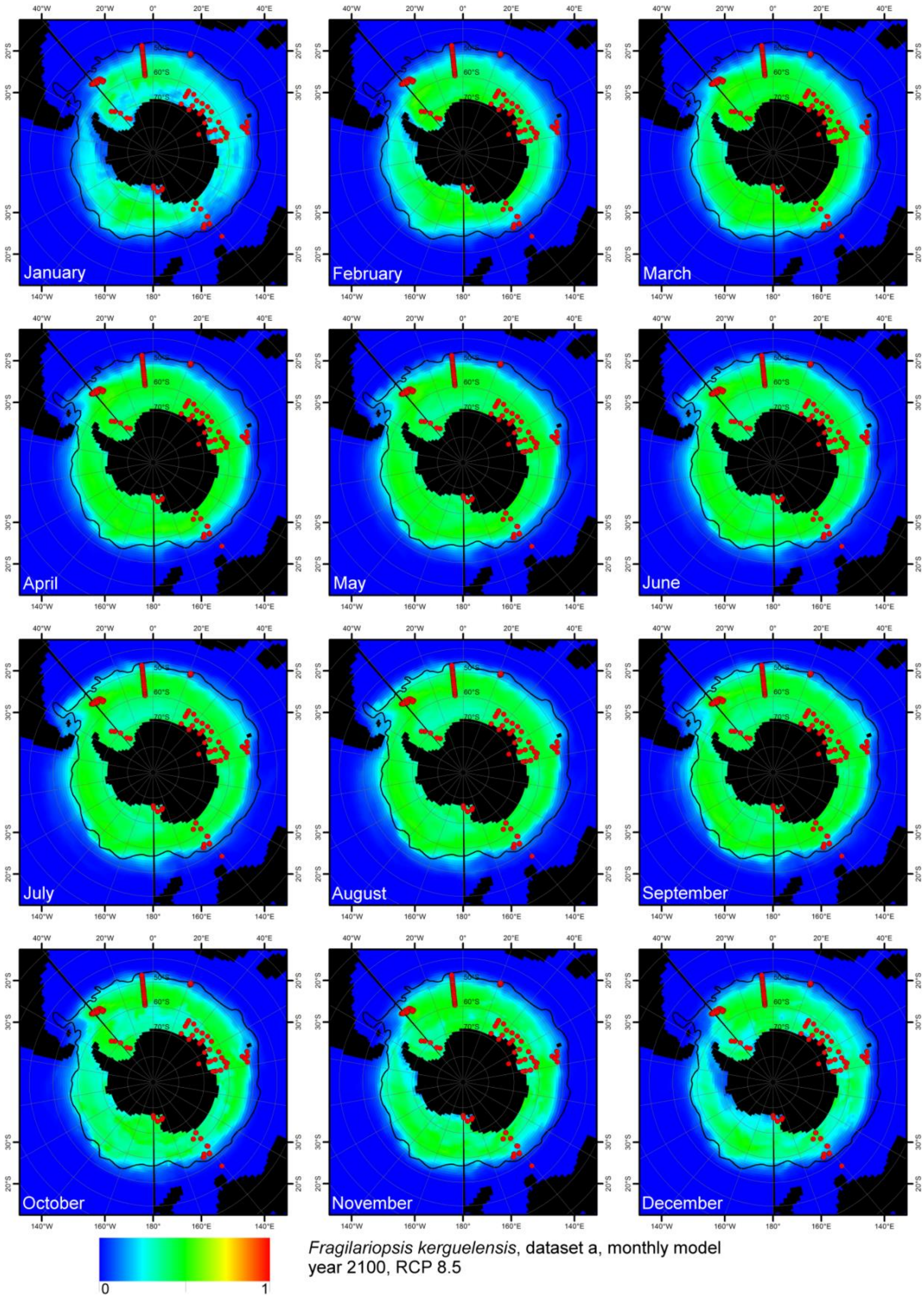

Fig. S3: Projections of the monthly Maxent model based on dataset A on environmental data predicted by climate models for the year 2100 for the RCP 4.5 scenario. The plots show the mean of the projections to five different climate model outputs. Average position of the Subantarctic Front is shown in black, observation points upon which the model is based are marked by red dots.

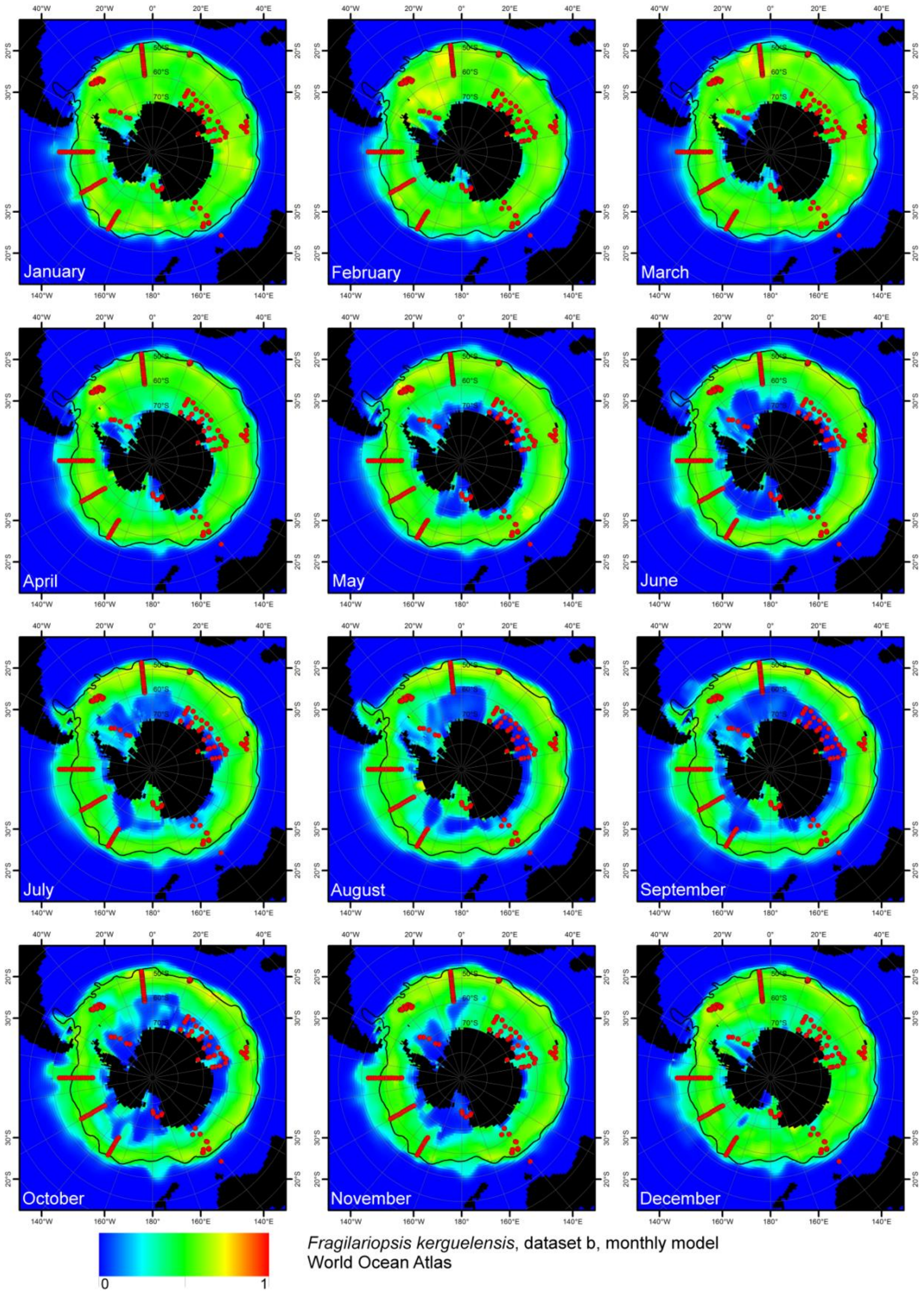

Fig. S4: Projections of the monthly Maxent model based on dataset B on monthly environmental data of the World Ocean Atlas in the course of the year. Average position of the Subantarctic Front is shown in black, observation points upon which the model is based are marked by red dots.

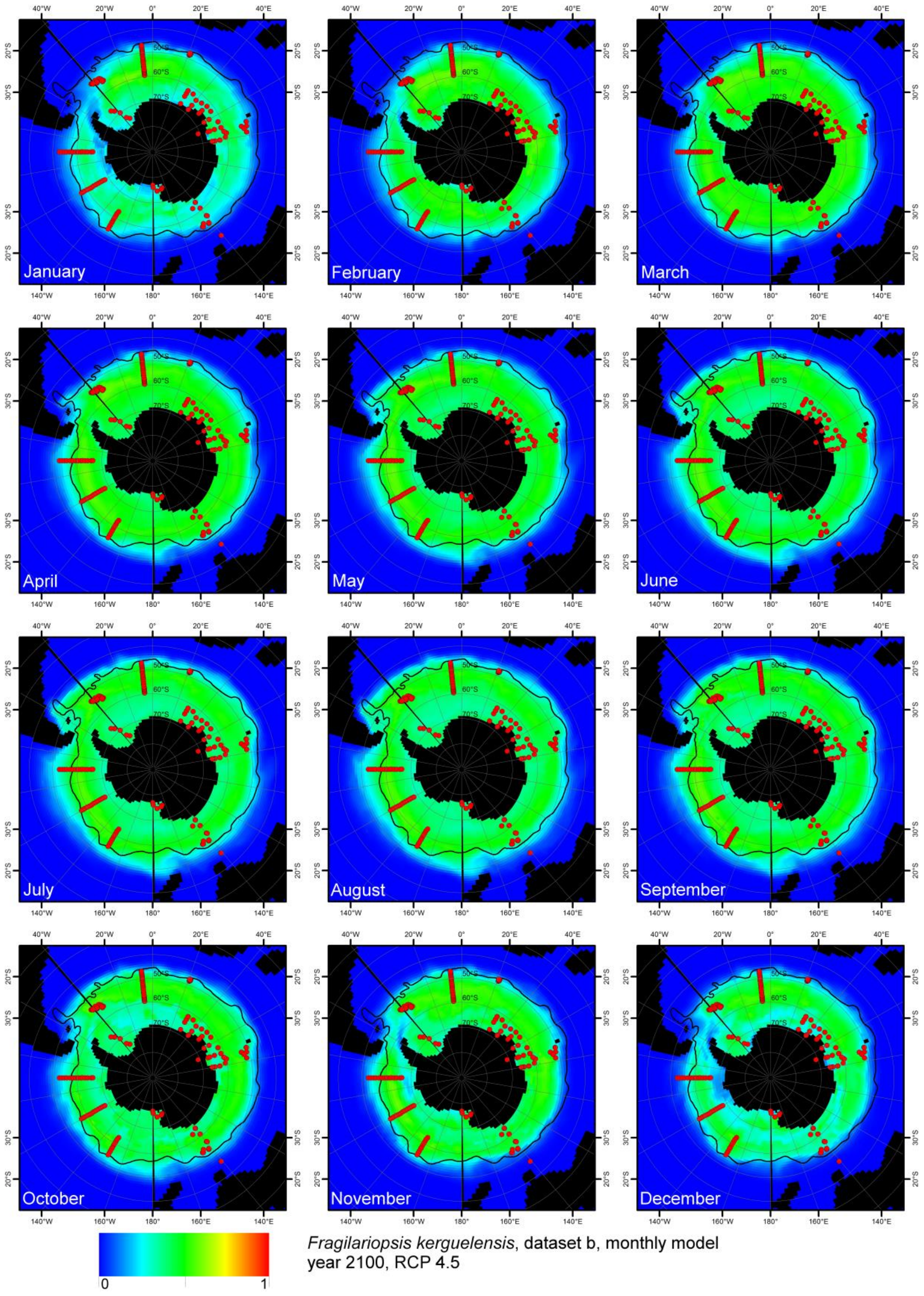

Fig. S5: Projections of the monthly Maxent model based on dataset B on environmental data predicted by climate models for the year 2100 for the RCP 4.5 scenario. The plots show the mean of the projections to five different climate model outputs. Average position of the Subantarctic Front is shown in black, observation points upon which the model is based are marked by red dots.

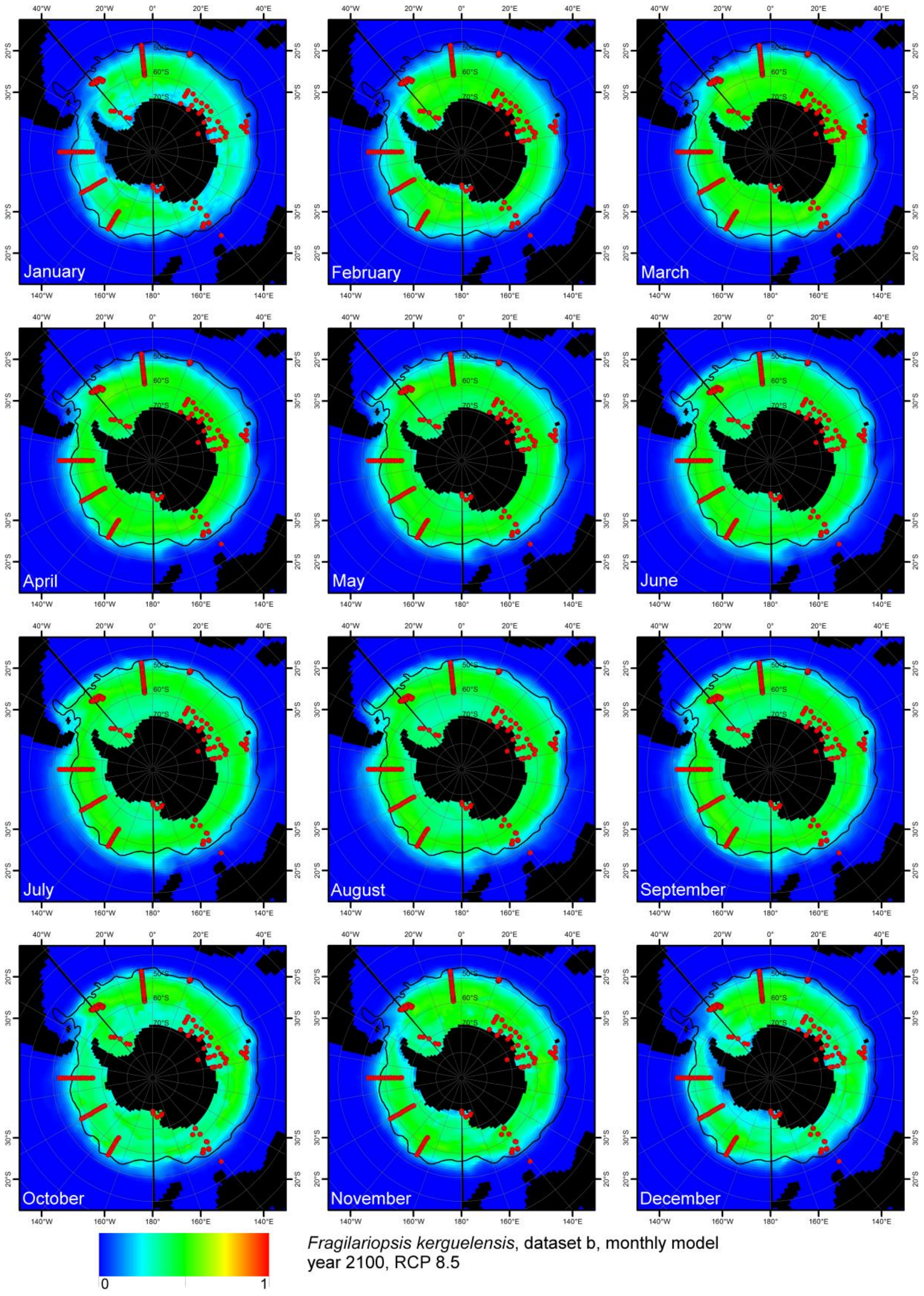

Fig. S6: Projections of the monthly Maxent model based on dataset B on environmental data predicted by climate models for the year 2100 for the RCP 8.5 scenario. The plots show the mean of the projections to five different climate model outputs. Average position of the Subantarctic Front is shown in red, observation points upon which the model is based are marked by red dots.

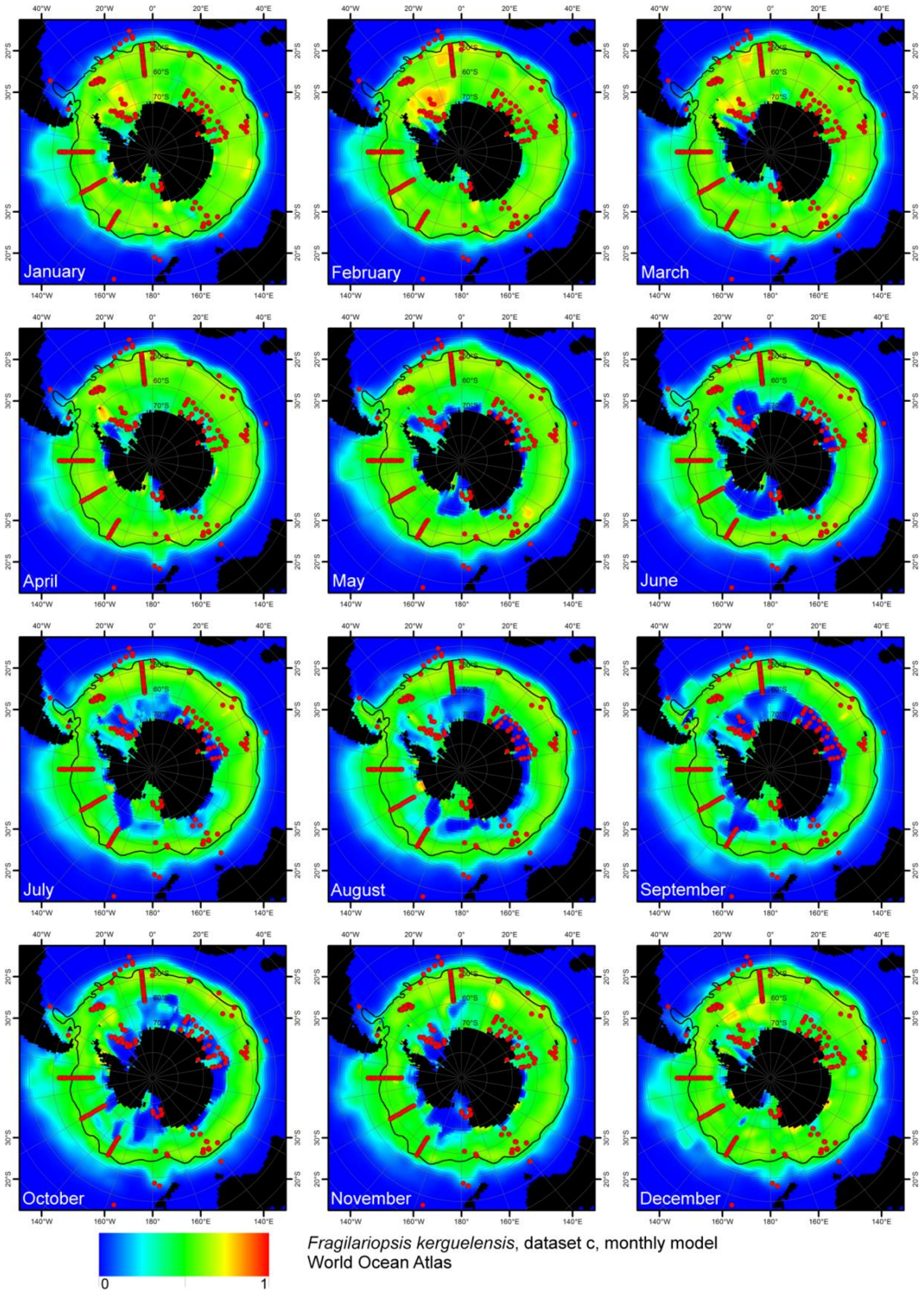

Fig. S7: Projections of the monthly Maxent model based on dataset C on monthly environmental data of the World Ocean Atlas in the course of the year. Average position of the Subantarctic Front is shown in black, observation points upon which the model is based are marked by red dots.

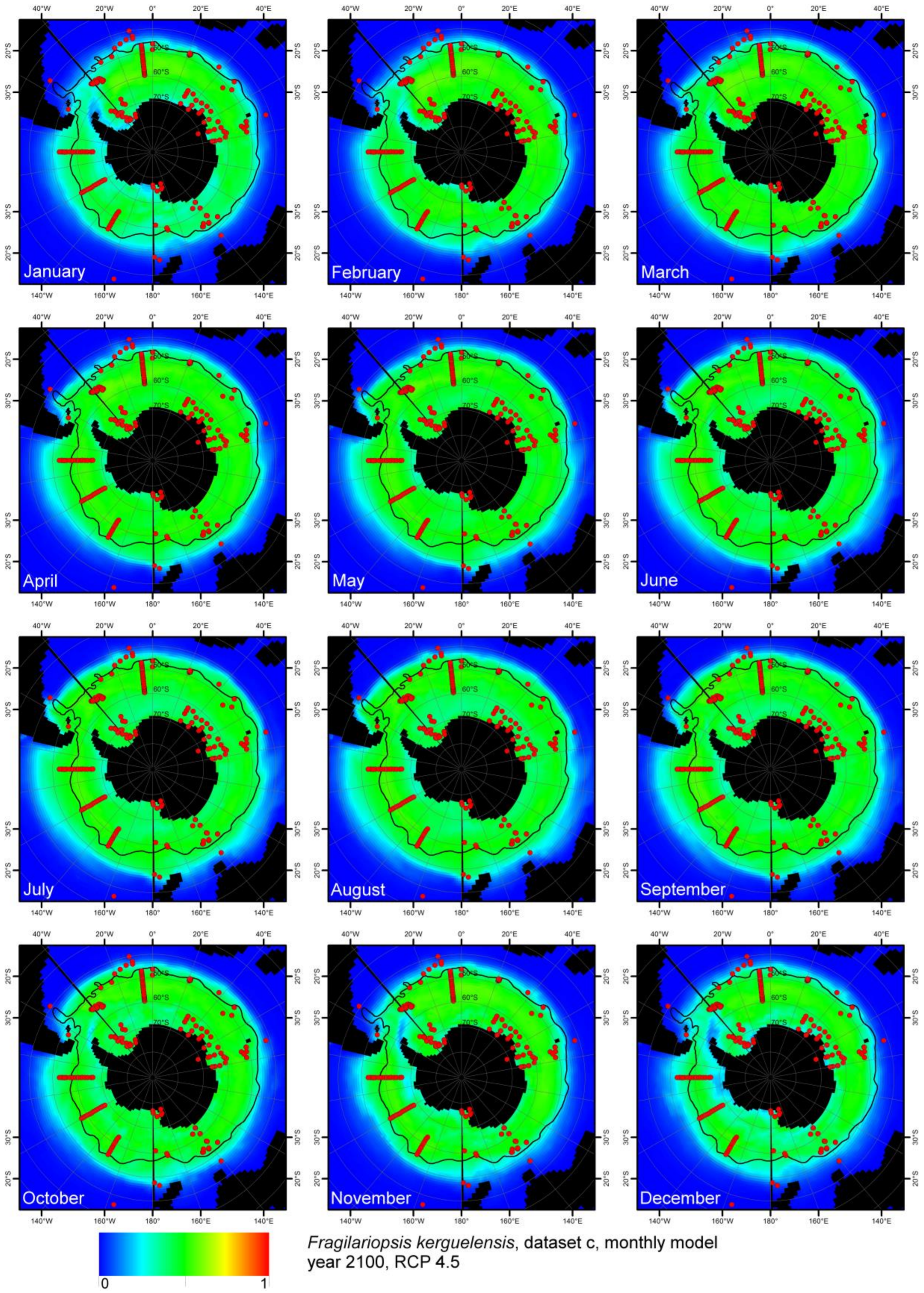

Fig. S8: Projections of the monthly Maxent model based on dataset C on monthly environmental data for year 2100, scenario RCP4.5 in the course of the year. Average position of the Subantarctic Front is shown in black, observation points upon which the model is based are marked by red dots.

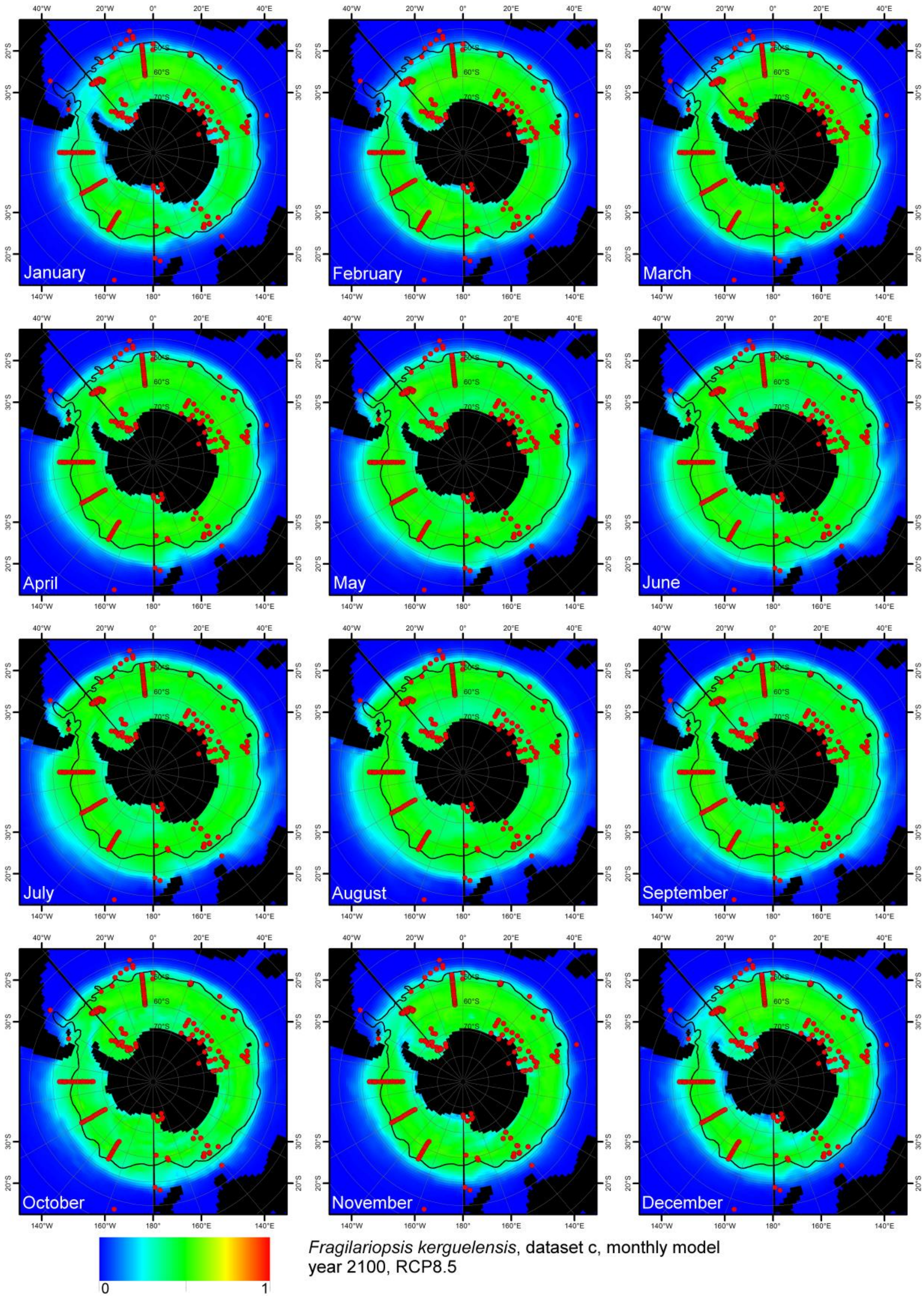

Fig. S9: Projections of the monthly Maxent model based on dataset C on monthly environmental data for year 2100, scenario RCP8.5 in the course of the year. Average position of the Subantarctic Front is shown in black, observation points upon which the model is based are marked by red dots.

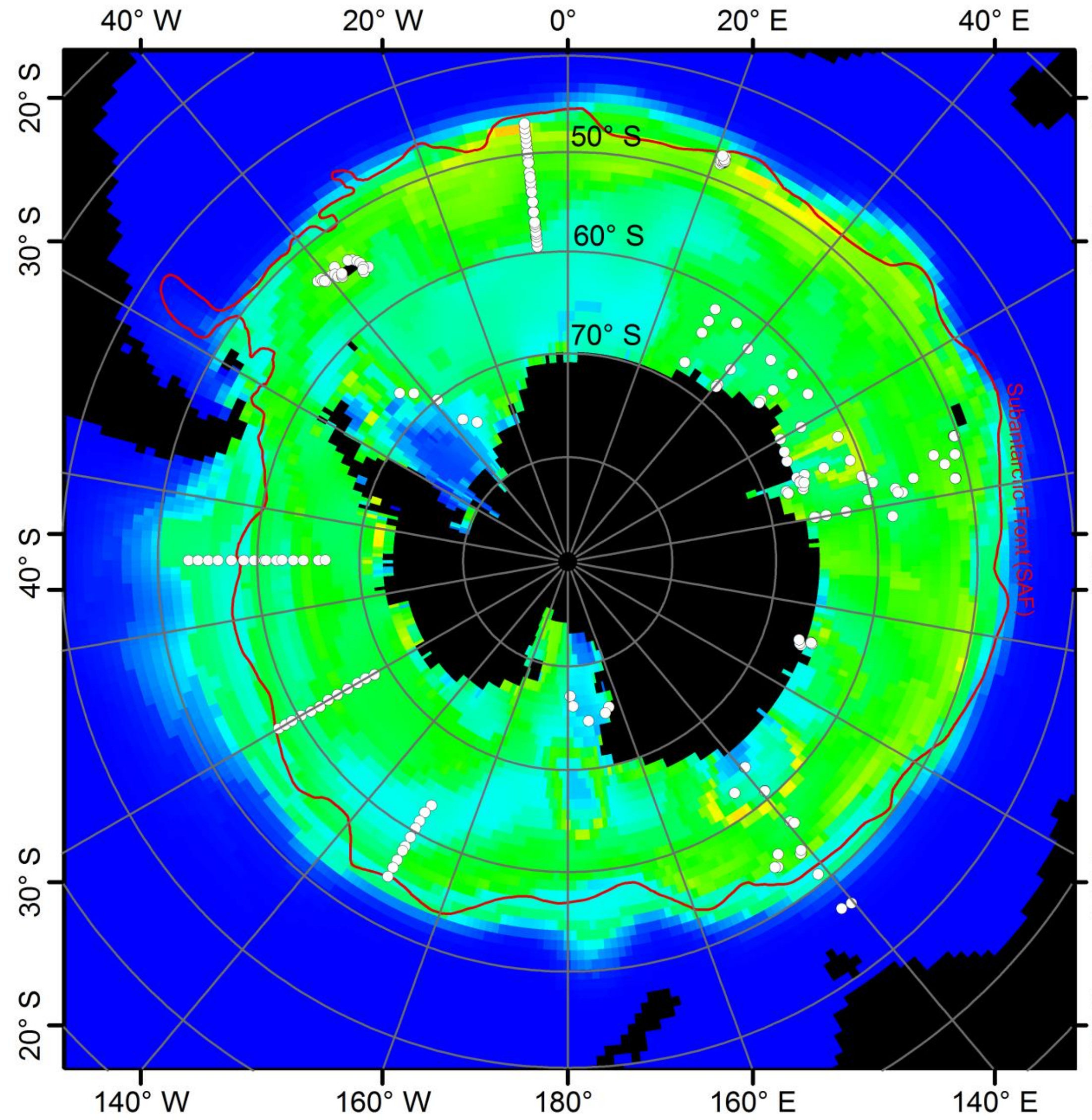

Fig. S10: Yearly Maxent model of *Fragilariopsis kerguelensis*, built using a bias-grid. The bias grid was based on all marine diatom (phylum Bacillariophyta*)* GBIF entries. A circle of 7° diameter was laid over each grid cell where an entry was located. Background points were generated by randomly selecting 1000 points from the regions covered by these circles for each month. This Maxent model was calculated with identical settings to the models presented in the main text.

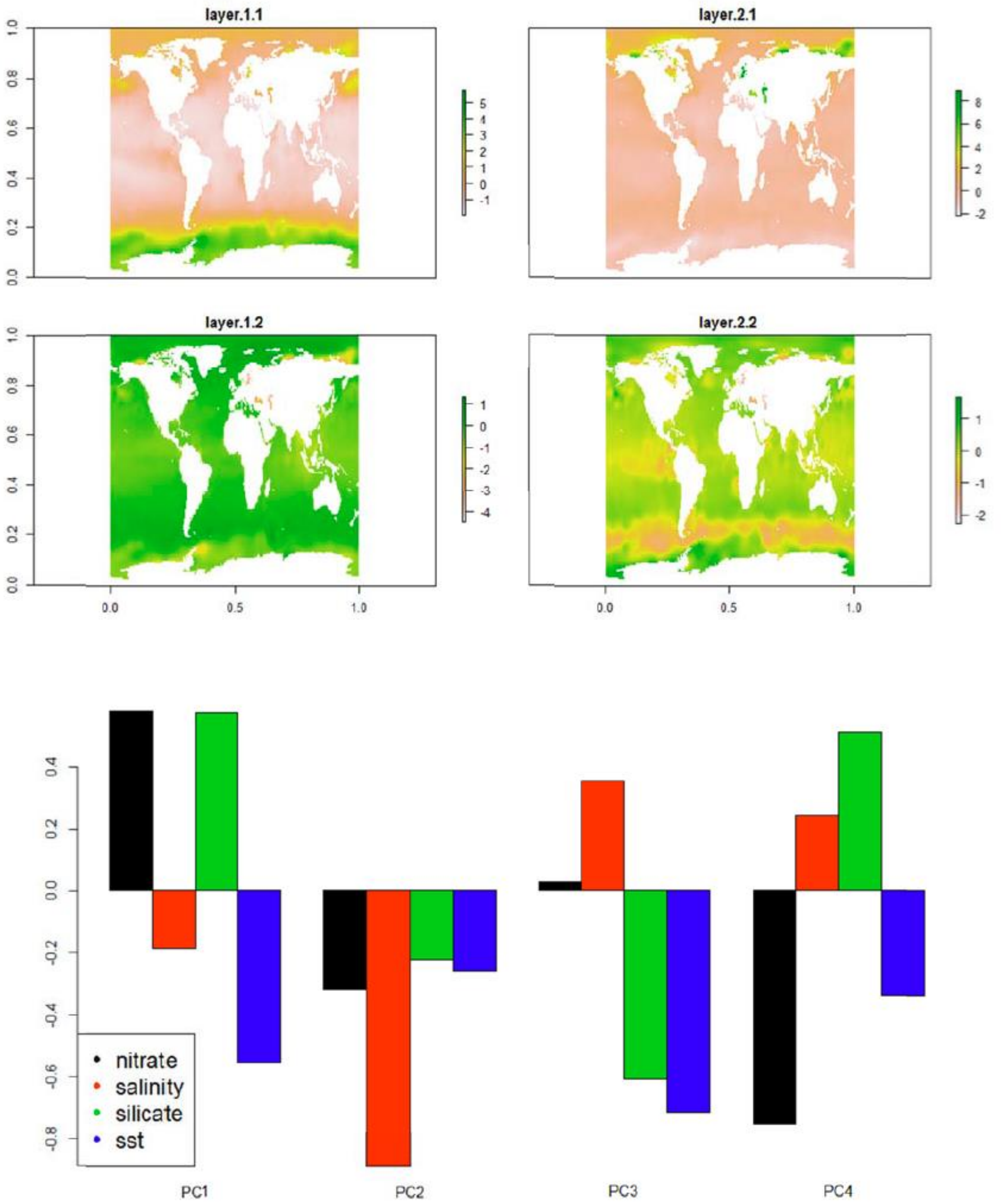

Fig. S11: Principal component analysis of the four environmental variables used as predictors in all analyses. Principal components were calculated from the correlation matrix of yearly average environmental data layers from WOA. The maps above show map projections of the four principal components; the bar plot below shows the variable loadings. The first principal component (PC1) represents an overall nutrient axis (see the high positive loadings of nitrate and silicate), whereas PC4 represents a nitrate vs. silicate contrast, illustrating that the generally high correlation between nitrate and silicate concentrations breaks down in the northern band of the Antarctic Circumpolar Current.

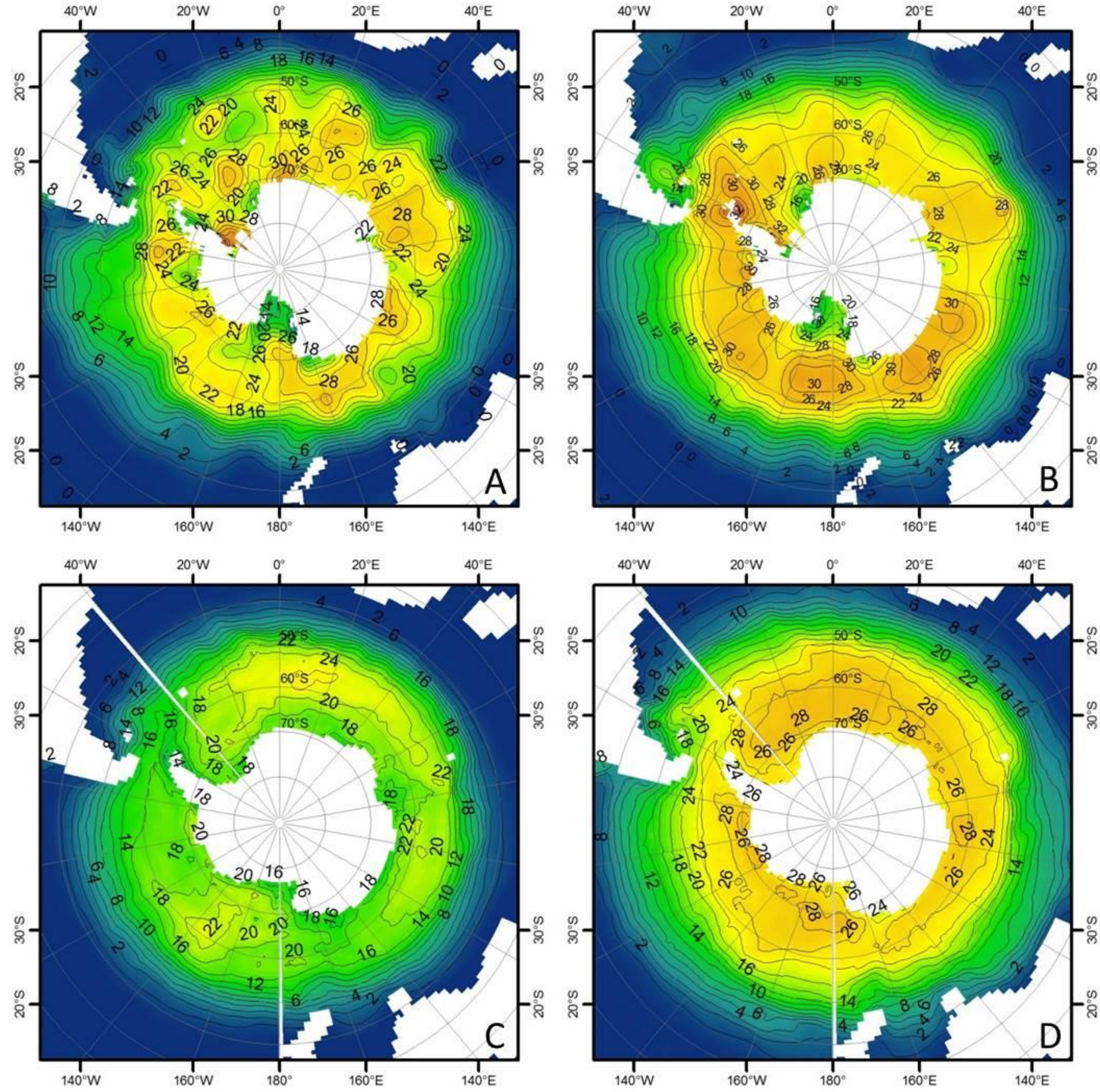

Fig. S12: Current and projected future sea surface nitrate distributions, A: Current, February World Ocean Atlas (WOA), B: Current, August, C: February 2100, RCP8.5, D: August 2100, RCP8.5. The plots illustrate that whereas summer peak nitrate concentrations are expected to decrease, areas with intermediate nitrate concentrations (10-20 µM, which still represent suitable conditions for F. kerguelensis) are expected to extend.

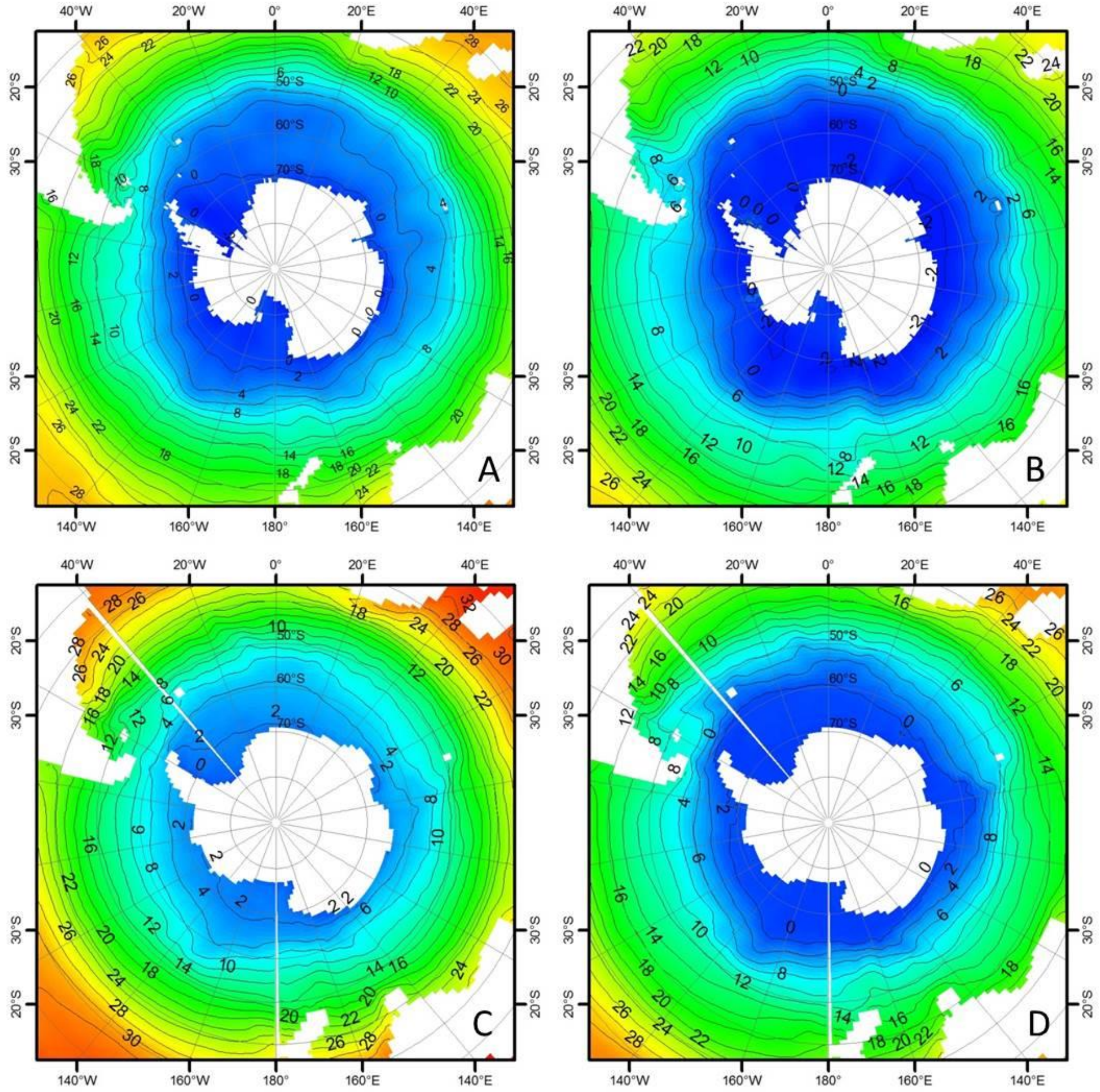

Fig. S13: Current and projected future seasonal distribution of sea surface temperature, A: Current, February, World Ocean Atlas (WOA), B: Current, August, C: February 2100, RCP8.5, D: August 2100, RCP8.5. The plots illustrate a general warming trend throughout the Southern Ocean (in the summer by nearly 2 °C).

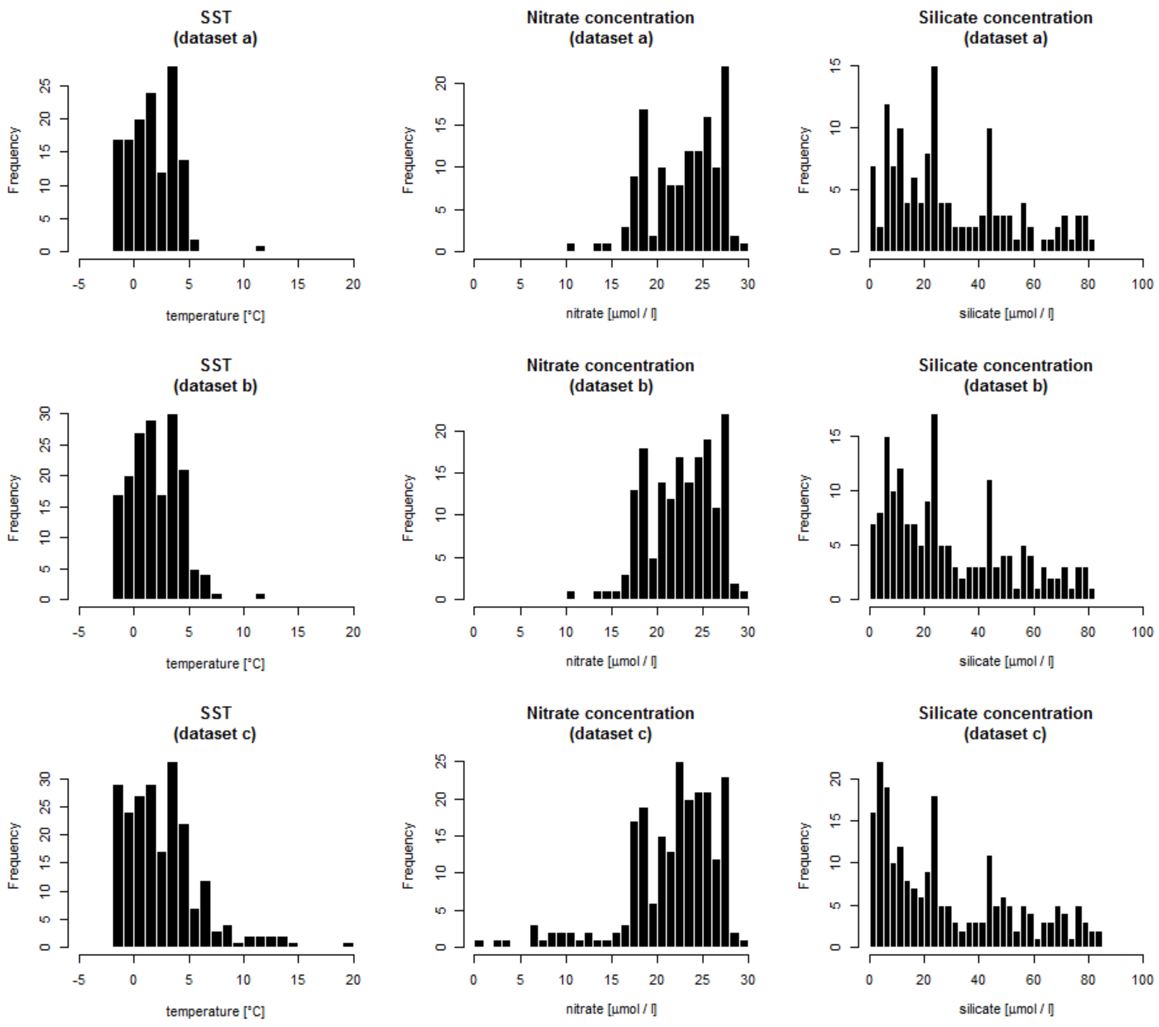

Fig. S14: Distribution of environmental conditions (sea surface temperature, nitrate, silicate – salinity is not shown due to its negligible influence in our models upon the distribution of *F. kerguelensis*) over the sampling sites for the three observation datasets.

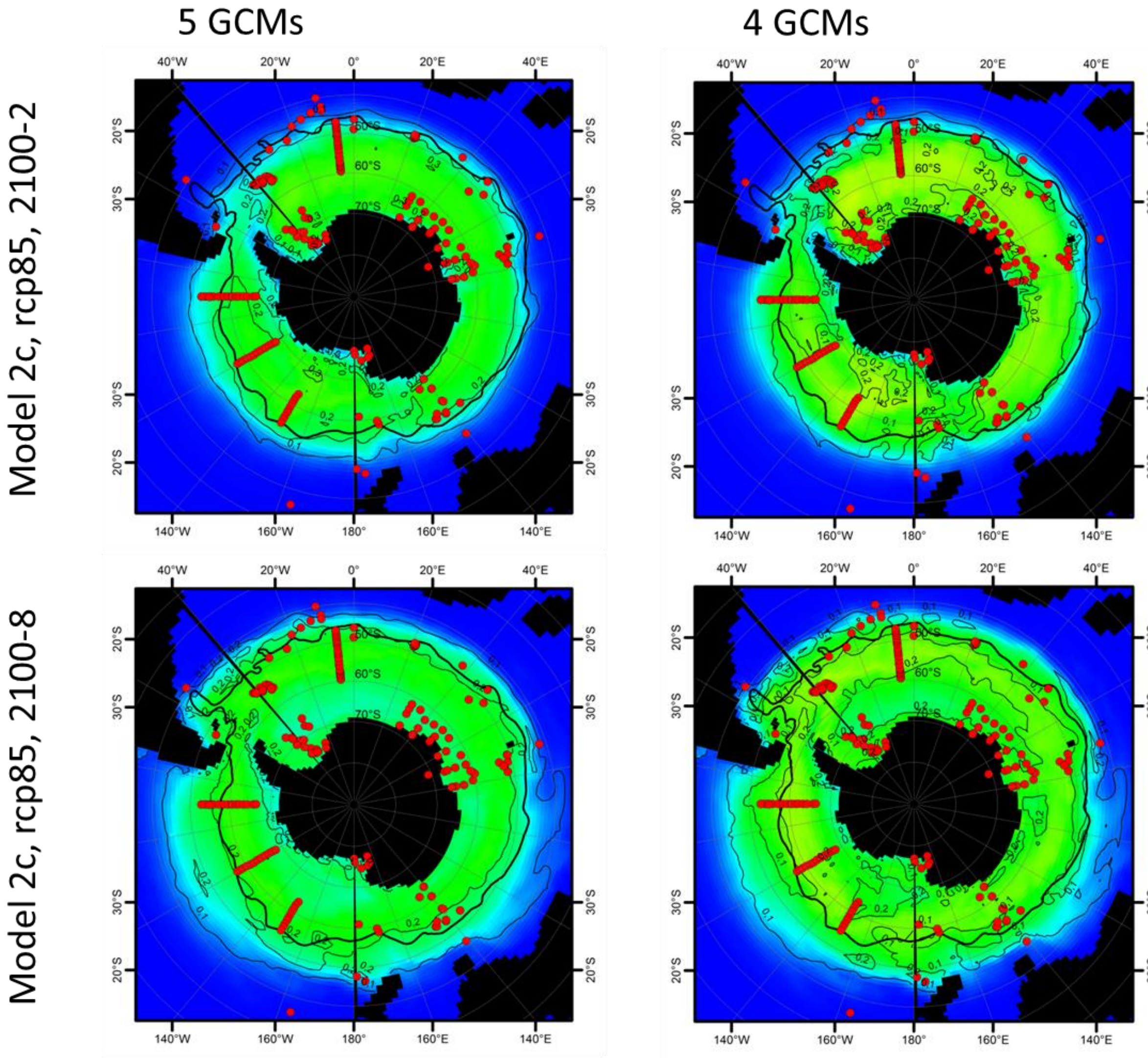

Fig. S15: Mean of the projection on only 4 (without CESM1-BGC model) vs. all 5 GCM outputs. The nitrate concentrations projected by CESM1-BGC markedly differ from those from the other four GCM outputs and also influence the northern distribution boundary projected for *F. kerguelensis*. The exclusion of CESM1-BGC does not substantially change the mean distribution area projections for *F. kerguelensis*, due to the relative consistency among the remaining four models. However, if the much lower nitrate projections of CESM1-BGC were nearer reality than those of the other models, this could lead to substantial shrinking of the *F. kerguelensis* distribution area (not shown).
